# Supplementary material for: Timing anthropogenic stressors to mitigate their impact on marine ecosystem resilience
Source: Nat Commun. 2017 Nov 2;8:1263. doi: 10.1038/s41467-017-01306-9 (PMC5665875; doi:10.1038/s41467-017-01306-9)
Supplement: Supplementary file 1 — Supplementary Information [file 41467_2017_1306_MOESM1_ESM.pdf]

Timing anthropogenic stressors to mitigate their impact on marine ecosystem resilience

Supplementary Information

Authors: Paul Pao-Yen Wu[12]*, Kerrie Mengersen[12], Kathryn McMahon[3,4], Gary A. Kendrick[4,5], Kathryn Chartrand[6], Paul H. York[6], Michael A. Rasheed[6], M. Julian Caley[1].

Supplementary Fig. 1 Recovery Time

Time to recovery of population (shoot density) for dredging scenarios applied to global sites. Each pie slice reflects the time to recovery for dredging starting in that month. Months are indicated by numerals in the outer ring where 12 denotes December. All results are aligned to Austral summers to enable seasonal comparisons. Pies are sorted row wise by genera then latitude from northernmost to southernmost; all 28 sites are shown for persistent *Amphibolis*, colonising *Halophila*, and opportunistic *Zostera*. Pies are sorted column wise by dredging duration from 0, 1, to 12 months. On the outer edge of each pie is the resilience criteria score (Method) ranging from dark green (all criteria satisfied), green, orange, yellow (loss but recovery within 6 months), to red for no criteria satisfied.

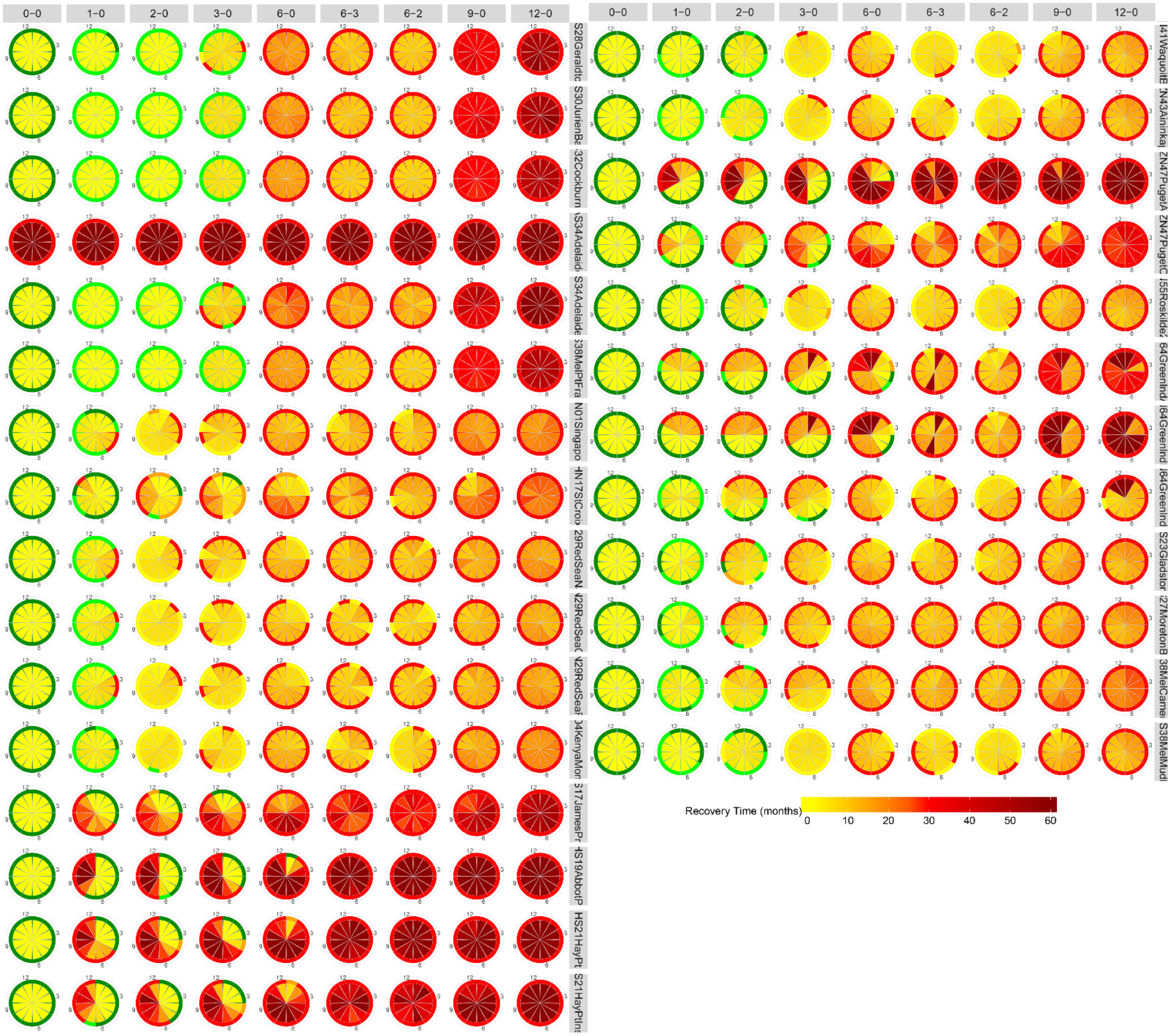
0–0
1–0
2–0
3–0
6–0
6–3
6–2
9–0
12–0
Recovery Time (months)
0
10
20
30
40
50
60

Supplementary Fig. 2 Extinction Risk

Ratio of the extinction risk compared to baseline for dredging scenarios applied to global sites. Each pie slice reflects this risk ratio for dredging starting in that month. Months are indicated by numerals in the outer ring where 12 denotes December. All results are aligned to Austral summers to enable seasonal comparisons. Pies are sorted row wise by genera then latitude from northernmost to southernmost; all 28 sites are shown for persistent *Amphibolis*, colonising *Halophila*, and opportunistic *Zostera*. Pies are sorted column wise by dredging duration from 0, 1, to 12 months. On the outer edge of each pie is the resilience criteria score (Method) ranging from dark green (all criteria satisfied), green, orange, yellow (loss but recovery within 6 months), to red for no criteria satisfied.

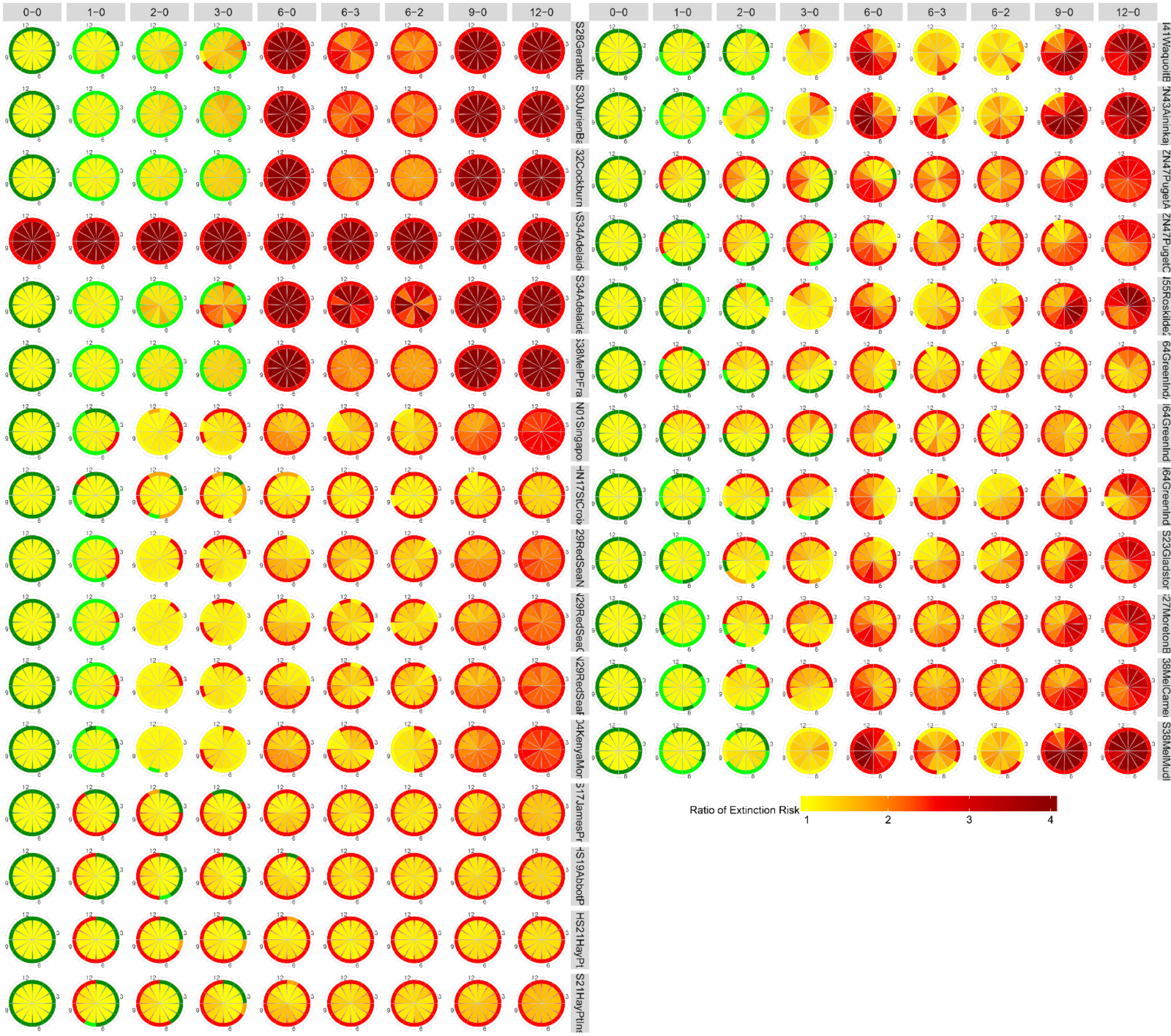
0–0
1–0
2–0
3–0
6–0
6–3
6–2
9–0
12–0
Ratio of Extinction Risk
1
2
3
4

Supplementary Fig. 3 Jurien Bay Scenario

Expected whole-of-system response to 2 months of dredging in February for persistent *Amphibolis* in Jurien Bay, Australia. Each group of panels represents an ecological or biological or environmental factor. Within each group, there are up to two panels where the top panel depicts the state probability trajectories over time for the states indicated and the bottom panel the weighted mean response where it is relevant. The initial 24 months prior to dredging are used for initialisation to allow the system to enter the baseline pattern (limit cycle, see Main Paper) and the grey region denotes the 2 month dredging period. Such a dredging campaign impacts the physiological status of the plant and imparts a loss in population measured here with shoot density; these have flow-on effects on factors such as the ability to resist further hazards, growth and recruitment and hence recovery, and ultimately the shoot density of the meadow (note increase in probability of zero and low shoot density states immediately after dredging). However, the impact is sufficiently small such that resilience criteria 1 relating to minimal loss is still met (Main Paper).

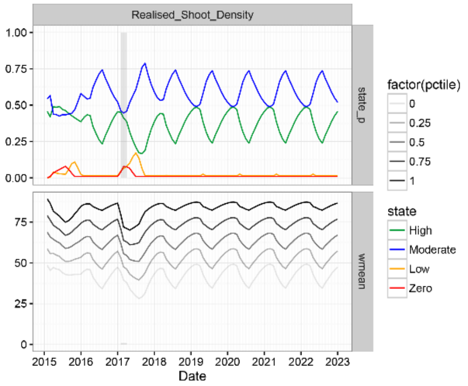
Realised_Shoot_Density
factor(pctile)
0
0.25
0.5
0.75
1
state
High
Moderate
Low
Zero
state_p
wmean
Date

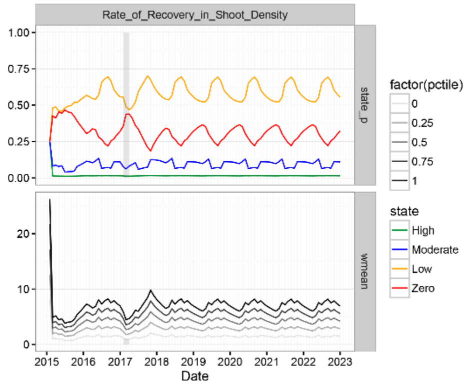
Rate_of_Recovery_in_Shoot_Density
factor(pctile)
0
0.25
0.5
0.75
1
state
High
Moderate
Low
Zero
state_p
wmean
Date

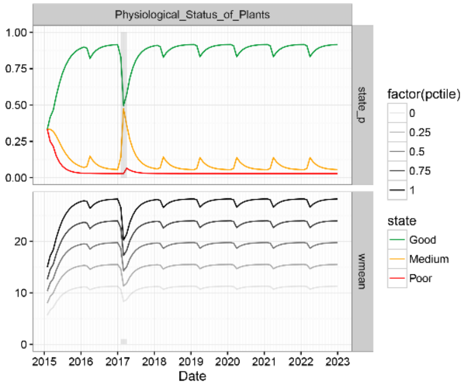
Physiological_Status_of_Plants
factor(pctile)
0
0.25
0.5
0.75
1
state
Good
Medium
Poor
state_p
wmean
Date

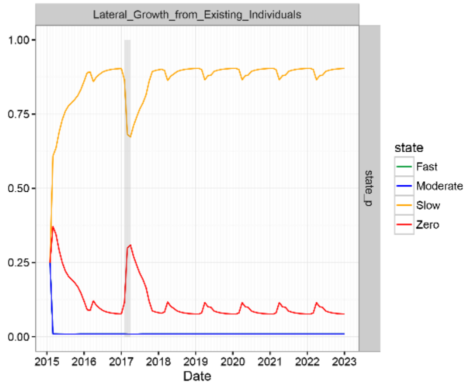
Lateral_Growth_from_Existing_Individuals
state
Fast
Moderate
Slow
Zero
state_p
Date

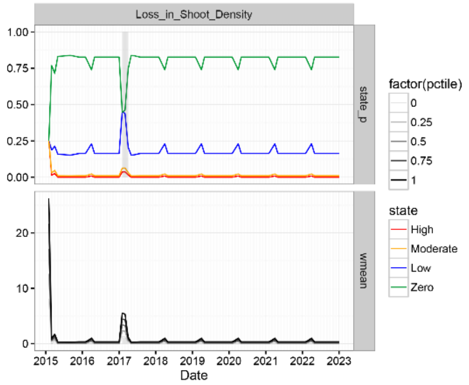
Loss_in_Shoot_Density
factor(pctile)
0
0.25
0.5
0.75
1
state
High
Moderate
Low
Zero
state_p
wmean
Date

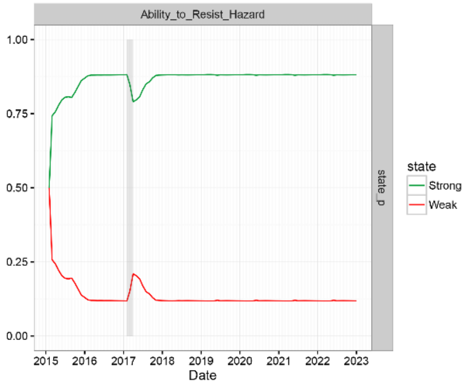
Ability_to_Resist_Hazard
state
Strong
Weak
state_p
Date

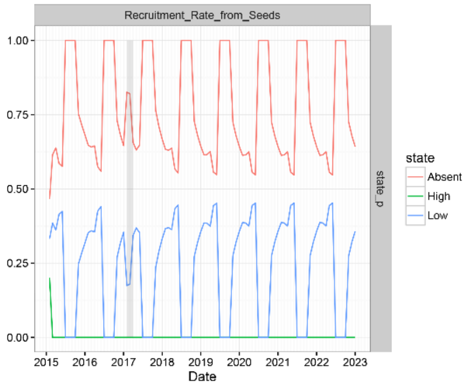
Recruitment_Rate_from_Seeds
state
Absent
High
Low
state_p
Date

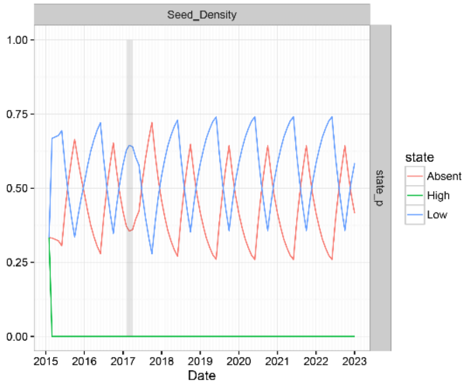
Seed_Density
state
Absent
High
Low
state_p
Date

Supplementary Fig. 4 Singapore Scenario

Expected whole-of-system response to 2 months of dredging in February for enduring, colonising *Halophila* in Singapore. Each group of panels represents an ecological or biological or environmental factor. Within each group, there are up to two panels where the top panel depicts the state probability trajectories over time for the states indicated and the bottom panel the weighted mean response where it is relevant. The initial 24 months prior to dredging are used for initialisation to allow the system to enter the baseline pattern (limit cycle, see Main Paper) and the grey region denotes the 2 month dredging window. Such dredging impacts the physiological status of the plant and imparts a loss in population measured here with shoot density; these have flow-on effects on factors such as the ability to resist further hazards, growth and recruitment and hence recovery, and ultimately the shoot density of the meadow (note increase in probability of zero and low shoot density states after dredging). Compared to Supplementary Fig. 3, note the significant probabilities for fast growth and high seed density of *Halohila* compared to slow growth and low seed for *Amphibolis*. They have much more pronounced seasonal variation in shoot density and other factors, and demonstrate less resistance with a marked drop due to dredging. However, they also recover rapidly which meets the second resilience criterion (Main Paper).

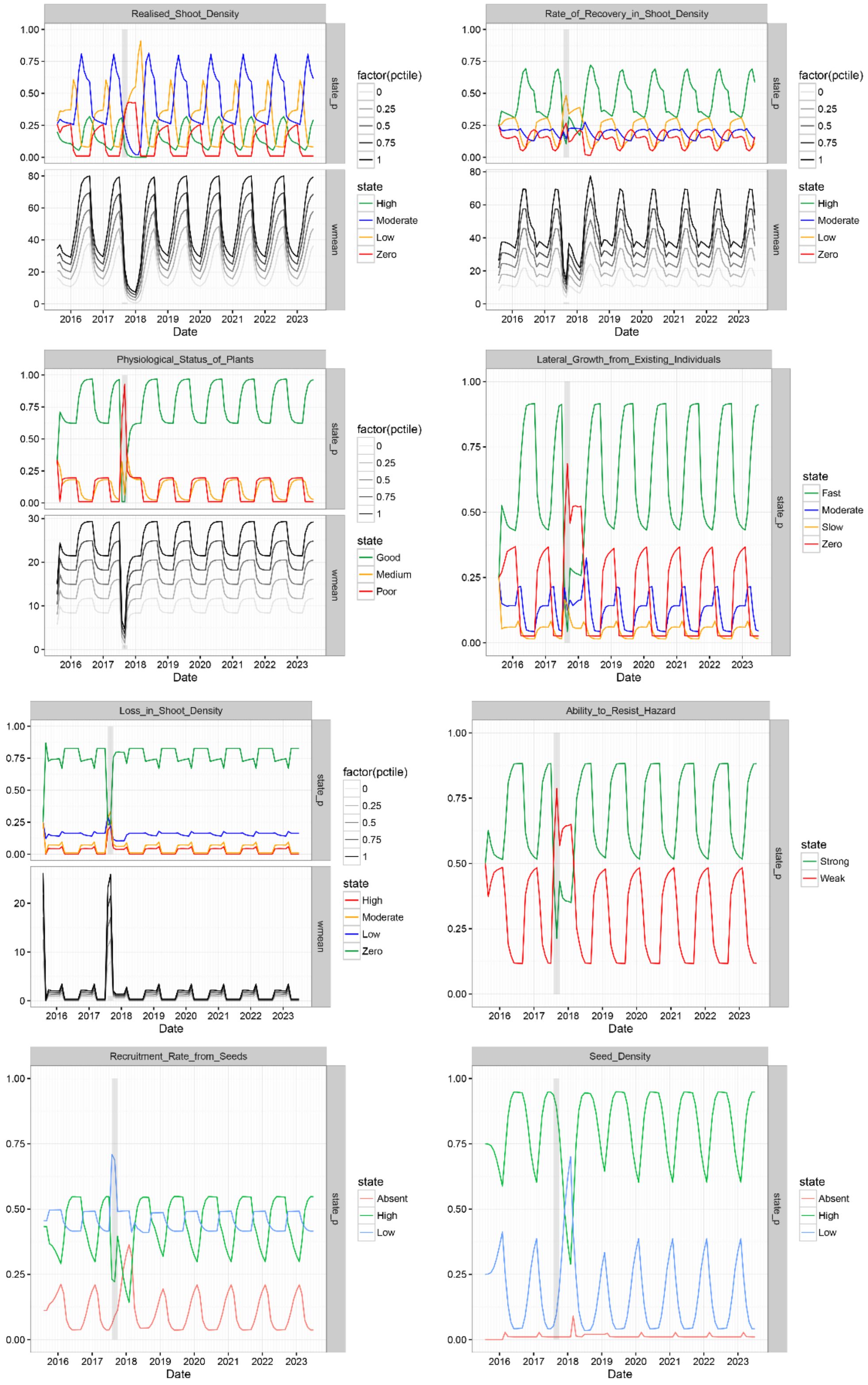
Realised_Shoot_Density
Rate_of_Recovery_in_Shoot_Density
Physiological_Status_of_Plants
Lateral_Growth_from_Existing_Individuals
Loss_in_Shoot_Density
Ability_to_Resist_Hazard
Recruitment_Rate_from_Seeds
Seed_Density
state_p
wmean
Date
2016 2017 2018 2019 2020 2021 2022 2023
factor(pctile)
0
0.25
0.5
0.75
1
state
High
Moderate
Low
Zero
Good
Medium
Poor
Fast
Slow
Strong
Weak
Absent

Supplementary Fig. 5 Aininkap Scenario

Expected whole-of-system response to 2 months of dredging in February for opportunistic *Zostera* in Aininkap, Japan. Each group of panels represents an ecological or biological or environmental factor. Within each group, there are up to two panels where the top panel depicts the state probability trajectories over time for the states indicated and the bottom panel the weighted mean response where it is relevant. The initial 24 months prior to dredging are used for initialisation to allow the system to enter the baseline pattern (limit cycle, see Main Paper) and the grey region denotes the 2 month dredging window. Such dredging impacts the physiological status of the plant and imparts a loss in population measured here with shoot density; these have flow-on effects on factors such as the ability to resist further hazards, growth and recruitment and hence recovery, and ultimately the shoot density of the meadow (note increase in probability of zero and low shoot density states after dredging). Compared to Supplementary Fig. 3 and 4, note the significant probabilities for fast growth and high seed density of *Zostera* similar to *Halohila* compared to slow growth and low seed for *Amphibolis*. They have less pronounced seasonal variation in shoot density and other factors than *Halophila*, and demonstrate more resistance with a lesser drop from dredging. However, this drop is still greater than that for *Amphibolis* when we compare probability of zero or low shoot density states. This combination of resistance and recovery enables opportunistic meadows to meet resilience criteria 1 and 2 respectively (Main Paper) for smaller stressors (e.g. dredging durations of up to approximately 2 months), and criteria 2 for larger stressors (e.g. longer dredging durations).

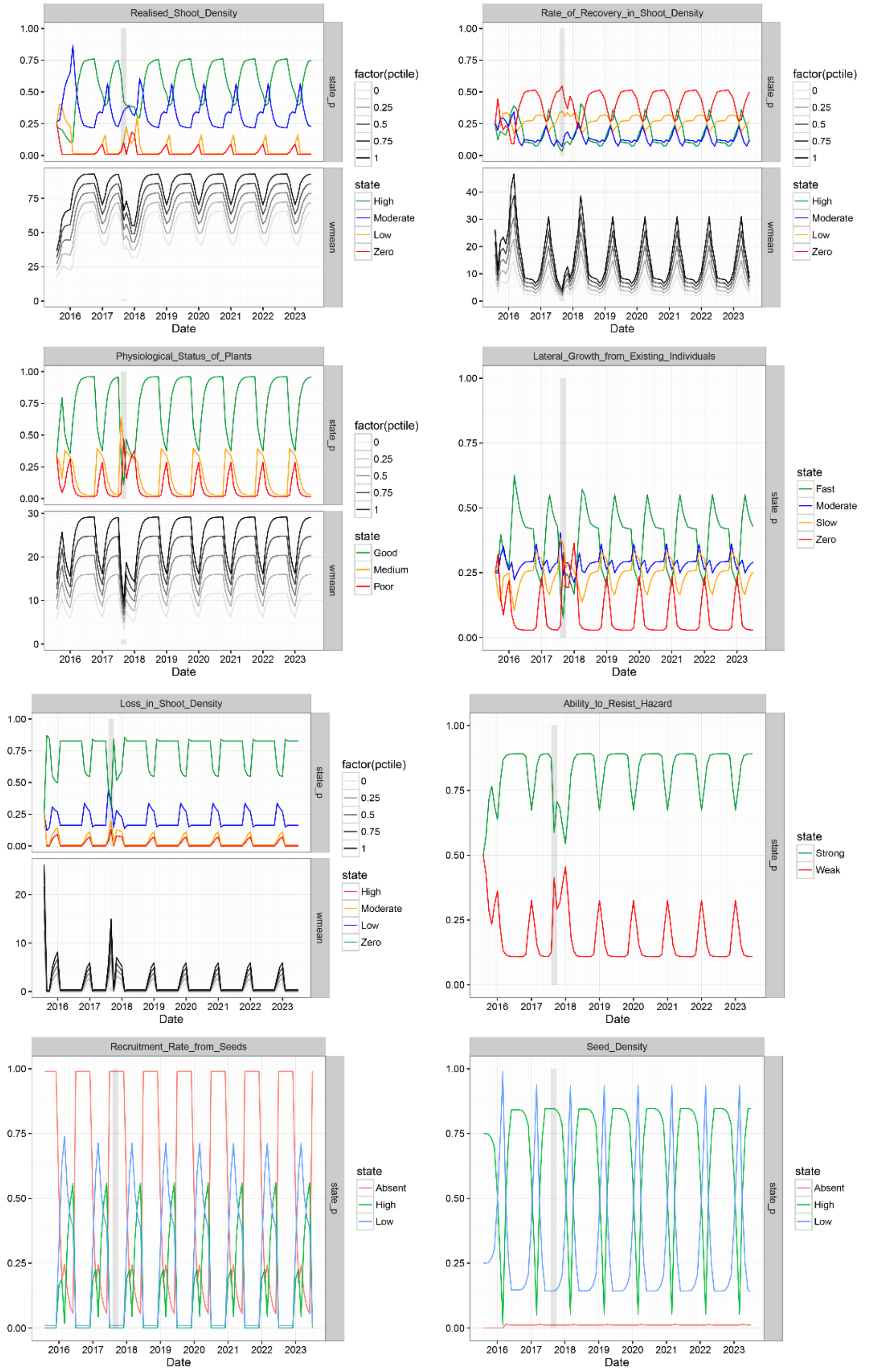
Realised_Shoot_Density
Rate_of_Recovery_in_Shoot_Density
Physiological_Status_of_Plants
Lateral_Growth_from_Existing_Individuals
Loss_in_Shoot_Density
Ability_to_Resist_Hazard
Recruitment_Rate_from_Seeds
Seed_Density
state_p
wmean
Date
factor(pctile)
0
0.25
0.5
0.75
1
state
High
Moderate
Low
Zero
Good
Medium
Poor
Fast
Slow
Strong
Weak
Absent
2016
2017
2018
2019
2020
2021
2022
2023

Supplementary Fig. 6 Kenya Scenario

Expected whole-of-system response to 2 months of dredging in February for enduring, colonising *Halophila* in Kenya. Each group of panels represents an ecological or biological or environmental factor. Within each group, there are up to two panels where the top panel depicts the state probability trajectories over time for the states indicated and the bottom panel the weighted mean response where it is relevant. The initial 24 months prior to dredging are used for initialisation to allow the system to enter the baseline pattern (limit cycle, see Main Paper) and the grey region denotes the 2 month dredging window. Such dredging impacts the physiological status of the plant and imparts a loss in population measured here with shoot density; these have flow-on effects on factors such as the ability to resist further hazards, growth and recruitment and hence recovery, and ultimately the shoot density of the meadow (note increase in probability of zero and low shoot density states after dredging). Compared to Supplementary Fig. 4, the state probability trajectories are very similar; however, the baseline variations in lateral growth and recruitment rate from seed are smaller which in part derives from shorter continuous periods of poorer light (Fig. 4, Main Paper). As a result, the recovery back to baseline is more rapid which enables this site to meet resilience criteria 2 (rapid recovery) and thus demonstrate greater resilience than the Singapore meadow. This is demonstrated in the ecological windows in Main Paper Fig. 3.

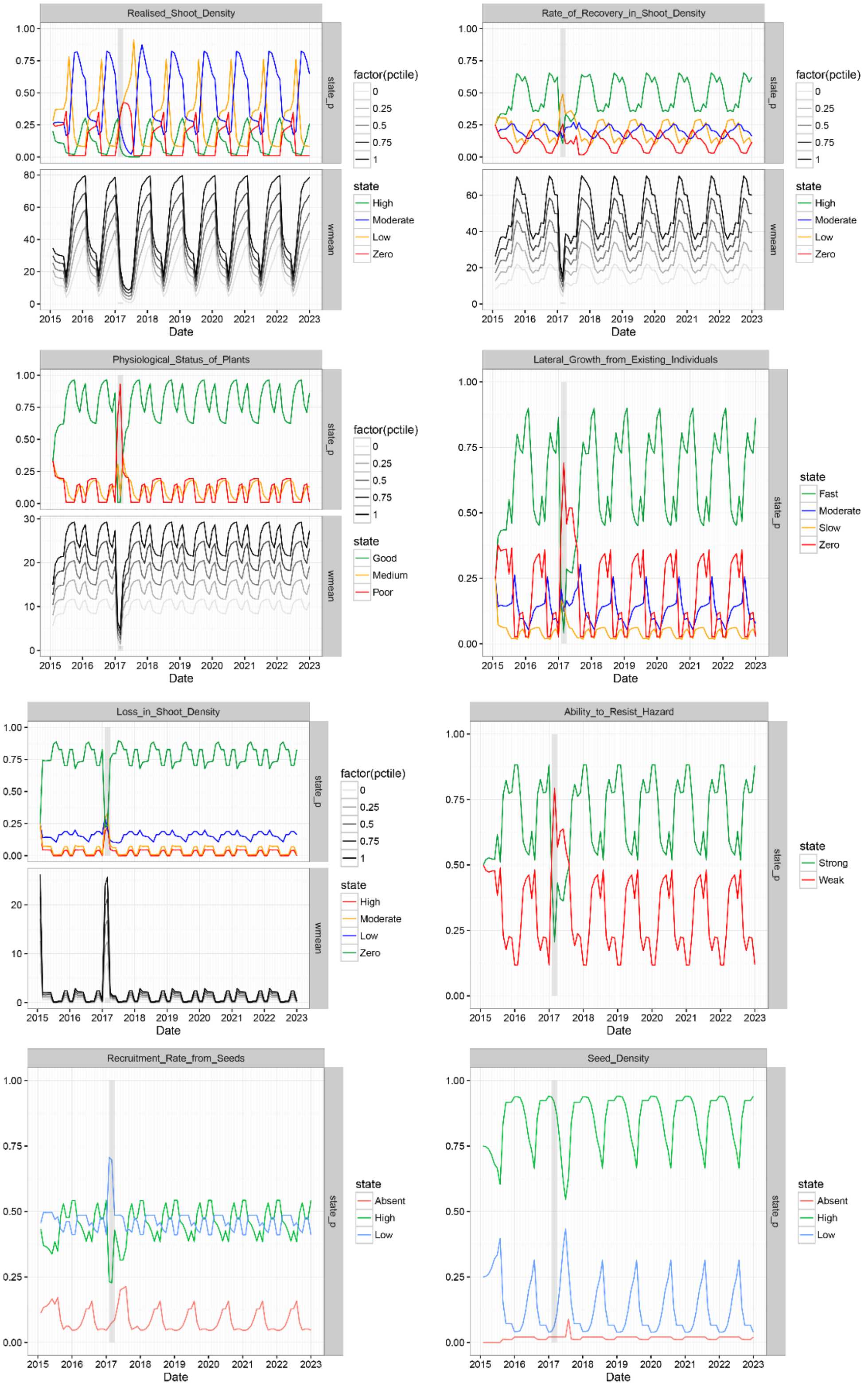
Realised_Shoot_Density
Rate_of_Recovery_in_Shoot_Density
Physiological_Status_of_Plants
Lateral_Growth_from_Existing_Individuals
Loss_in_Shoot_Density
Ability_to_Resist_Hazard
Recruitment_Rate_from_Seeds
Seed_Density
state_p
wmean
factor(pctile)
0
0.25
0.5
0.75
1
state
High
Moderate
Low
Zero
Good
Medium
Poor
Fast
Slow
Strong
Weak
Absent
Date
2015
2016
2017
2018
2019
2020
2021
2022
2023

Supplementary Fig. 7 Overall DBN

The overall Dynamic Bayesian Network (DBN) network structure. Ovals denote factors (also known as nodes), arrows denote causal parent-child relationships in the direction of the arc where a parent node (e.g. Meadow Type) influences a child node (e.g. Location Type); conversely, an absence of a link implies conditional independence[1]. Rounded rectangles denote subnetworks. Note the presence of complex interdependencies and feedback loops in the seagrass ecosystem. Although only shoot density is shown as one of the three main management indicators, biomass and aerial extent are connected in a similar way but omitted for clarity. The recovery, resistance and environment subnetworks are expanded in Supplementary Fig. 8 through 10 respectively. Note that each node could be backed by another model such as the case for accumulated light (Methods). In addition, all environmental nodes are either inputs or backed by an external model, hence they are not connected to each other in any way. Nodes are coloured as follows: white for input nodes, purple for recovery nodes, green for resistance nodes, blue for environmental nodes, yellow for population (shoot density) nodes, and pink for all other nodes.

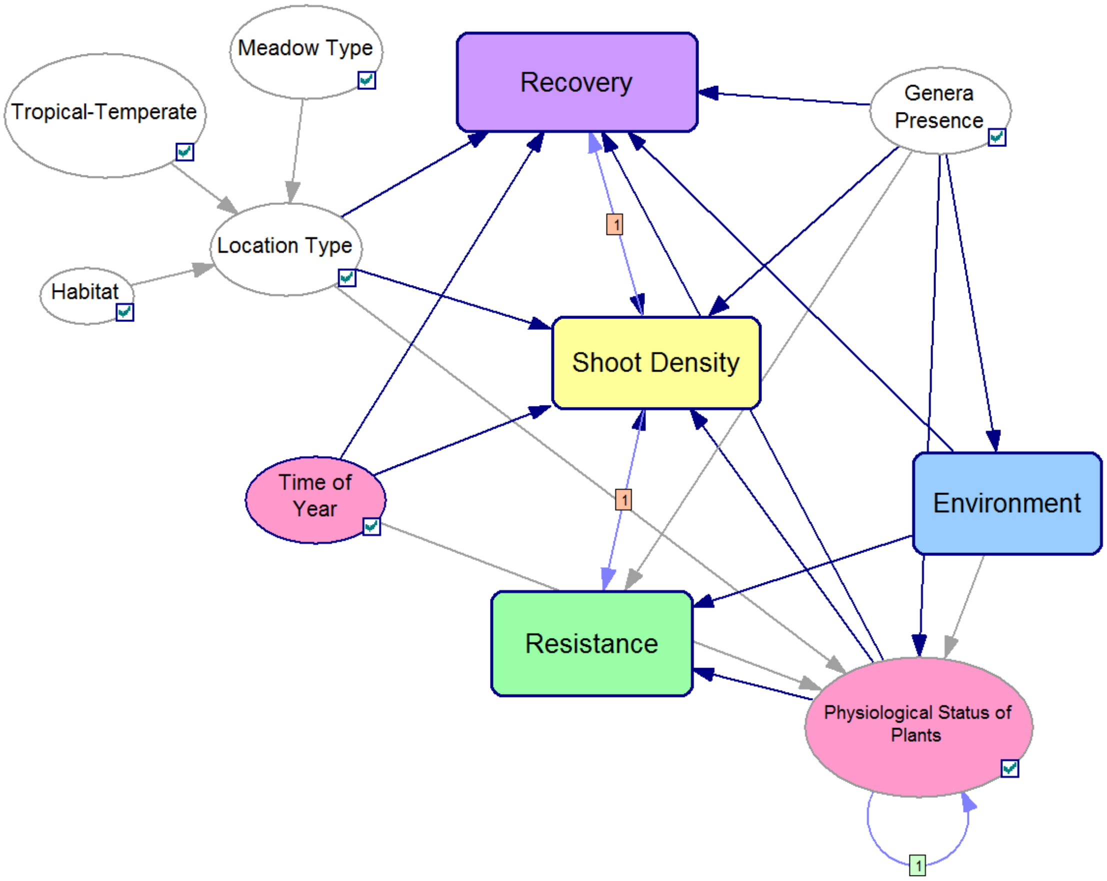
Meadow Type
Recovery
Genera Presence
Tropical-Temperate
Location Type
Habitat
1
Shoot Density
Time of Year
1
Environment
Resistance
Physiological Status of Plants
1

Supplementary Fig. 8 Recovery DBN

The Dynamic Bayesian Network (DBN) network structure for the recovery subnetwork. Ovals denote factors (also known as nodes), arrows denote conditional parent-child relationships where a parent node (e.g. Meadow Type) influences a child node (e.g. Location Type). Conversely, an absence of a link implies conditional independence. A triangle marking on the left or right of a node denotes a connection from or to another subnetwork. Nodes are coloured as follows: purple for recovery nodes, blue for environmental nodes, yellow for population (shoot density) nodes, and pink for all other nodes. Seed Density has parents Time of Year and Location Type in other subnetworks. Similarly, Recruitment Rate from Seeds has Accumulated Light, Accumulated Burial and Time of Year as parents.

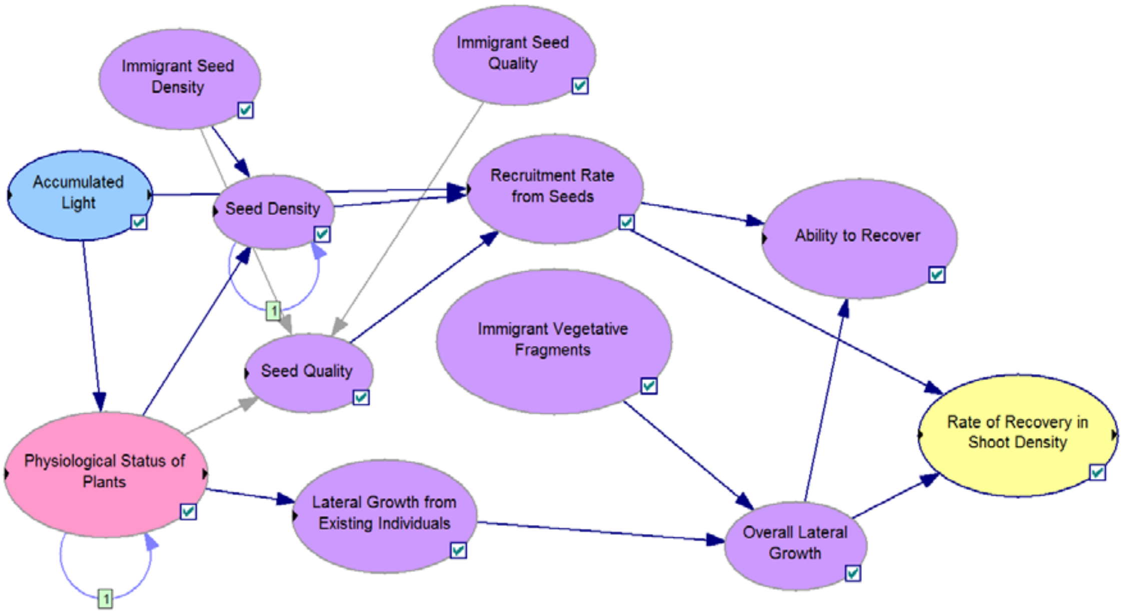

Supplementary Fig. 9 Loss and Resistance DBN

The Dynamic Bayesian Network (DBN) network structure for the loss and resistance subnetworks. Ovals denote factors (also known as nodes), arrows denote conditional parent-child relationships where a parent node (e.g. Meadow Type) influences a child node (e.g. Location Type). Conversely, an absence of a link implies conditional independence. A triangle marking on the left or right of a node denotes a connection from or to another subnetwork. Nodes are coloured as follows: green for resistance nodes, blue for environmental nodes, yellow for population (shoot density) nodes, and pink for all other nodes. From other subnetworks, Realised Shoot Density is a parent to both Ability to Resist and Above to Below Ground Biomass nodes; Genera Presence is also a parent to the latter. Physiological Status of Plants has parent nodes Genera Presence, Time of Year and Location Type.

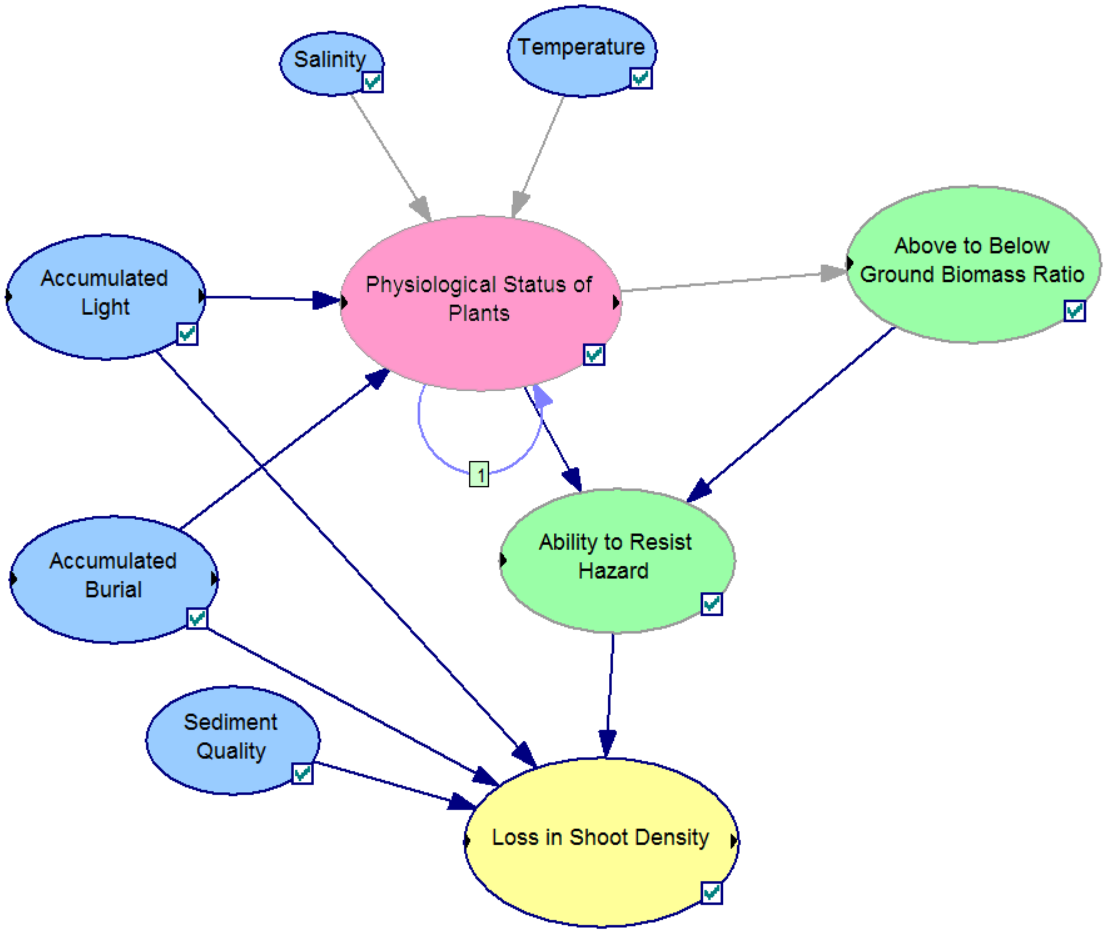
Salinity
Temperature
Accumulated Light
Physiological Status of Plants
Above to Below Ground Biomass Ratio
1
Accumulated Burial
Ability to Resist Hazard
Sediment Quality
Loss in Shoot Density

Supplementary Fig. 10 Environmental Inputs

The Dynamic Bayesian Network (DBN) network structure for the environmental inputs subnetwork. Ovals denote factors (also known as nodes). A triangle marking on the left or right of a node denotes a connection from or to another subnetwork. As these nodes are treated as inputs to the model, there are no connections between them. Nodes are coloured blue as they are environmental nodes.

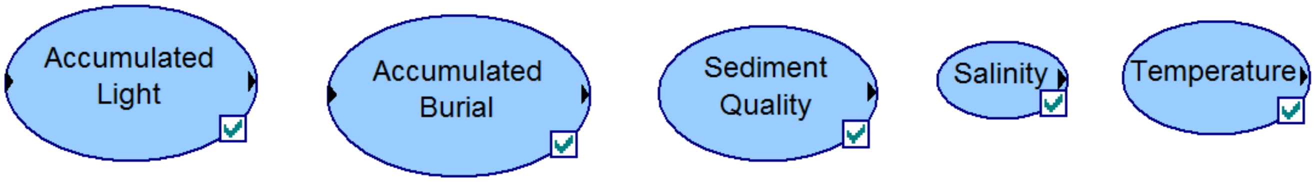

Supplementary Fig. 11 Shoot Density

The Dynamic Bayesian Network (DBN) network structure for the shoot density subnetwork showing connections to some other key nodes (note that biomass has the same structure). Ovals denote factors (also known as nodes). A triangle marking on the left or right of a node denotes a connection from or to another subnetwork. Nodes are coloured as follows: white for input nodes, purple for recovery nodes, green for resistance nodes, blue for environmental nodes, yellow for population (shoot density) nodes, and pink for all other nodes.

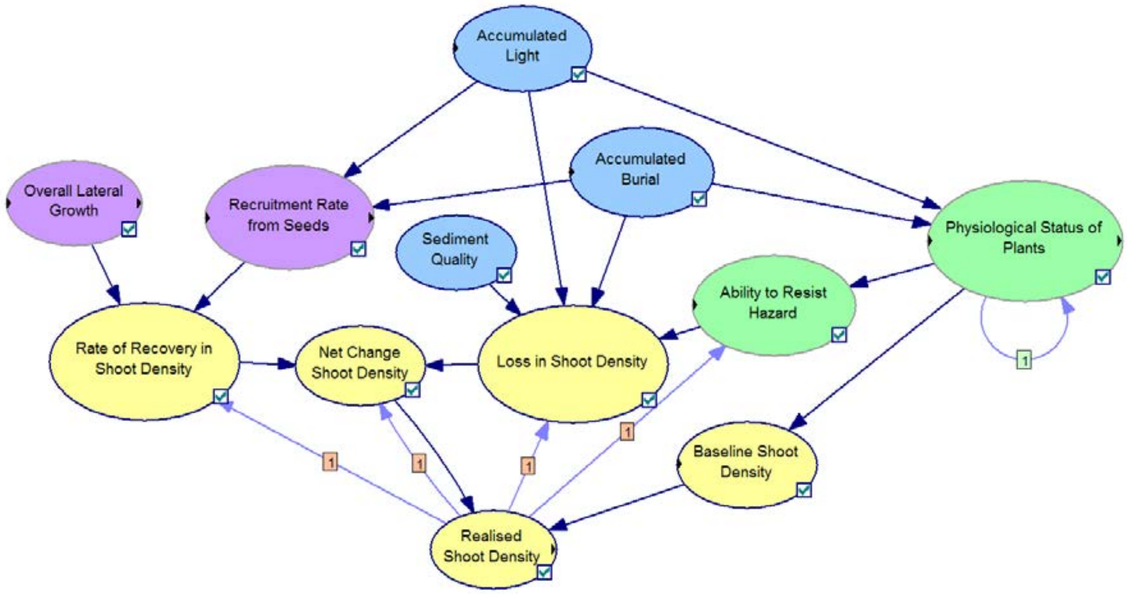

Supplementary Fig. 12 Ecological windows with 25% light during dredging

Ecological windows for seagrasses by life history strategy/genus, and dredging design for 25% probability of above saturation light during dredging. Each ring on a pie corresponds to a site, ordered from southernmost to northernmost going from innermost ring to outermost ring. Resistance, recovery and persistence criteria were considered. Criteria scores are colour coded: dark green for satisfaction of all criteria, light green for resistance and recovery criteria satisfied, orange for recovery and persistence, yellow for just recovery, and red for no criteria satisfied. A score that is not red is considered an ecological window.

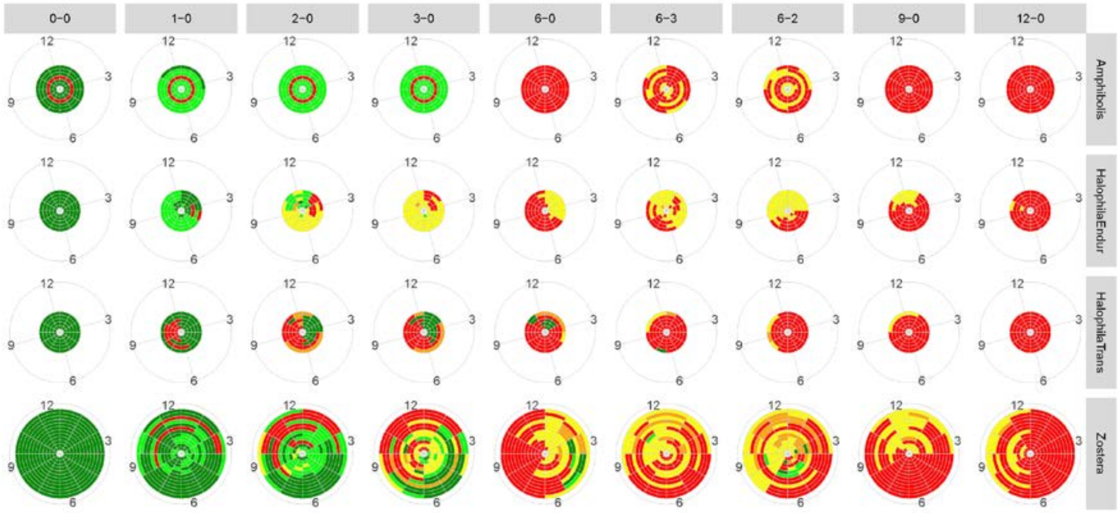

Supplementary Fig. 13 Ecological windows with 50% light during dredging

Ecological windows for seagrasses by life history strategy/genus, and dredging design for 50% probability of above saturation light during dredging. Each ring on a pie corresponds to a site, ordered from southernmost to northernmost going from innermost ring to outermost ring. Resistance, recovery and persistence criteria were considered. Criteria scores are colour coded: dark green for satisfaction of all criteria, light green for resistance and recovery criteria satisfied, orange for recovery and persistence, yellow for just recovery, and red for no criteria satisfied. A score that is not red is considered an ecological window.

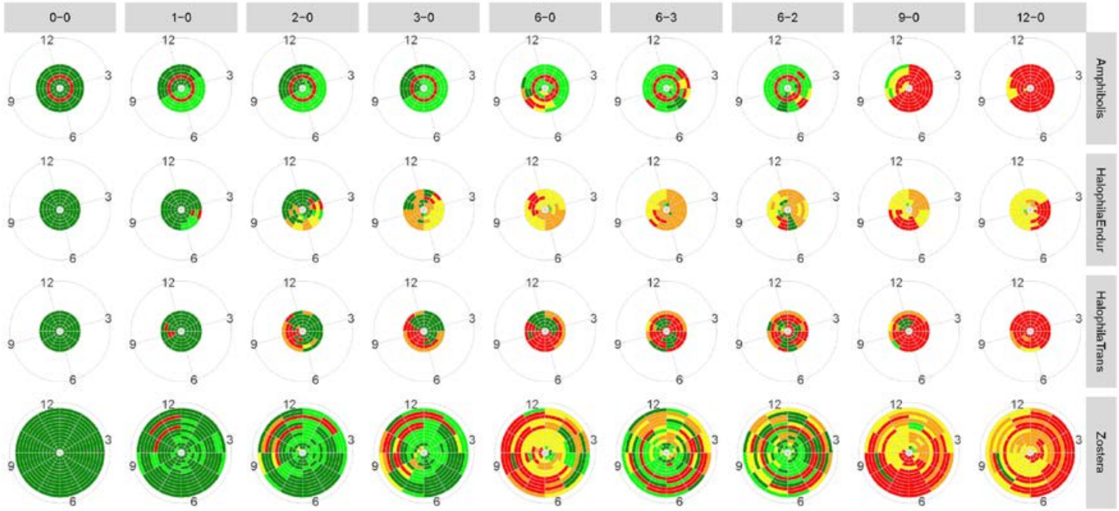

Supplementary Fig. 14 Ecological windows with 75% light during dredging

Ecological windows for seagrasses by life history strategy/genus, and dredging design for 75% probability of above saturation light during dredging. Each ring on a pie corresponds to a site, ordered from southernmost to northernmost going from innermost ring to outermost ring. Resistance, recovery and persistence criteria were considered. Criteria scores are colour coded: dark green for satisfaction of all criteria, light green for resistance and recovery criteria satisfied, orange for recovery and persistence, yellow for just recovery, and red for no criteria satisfied. A score that is not red is considered an ecological window.

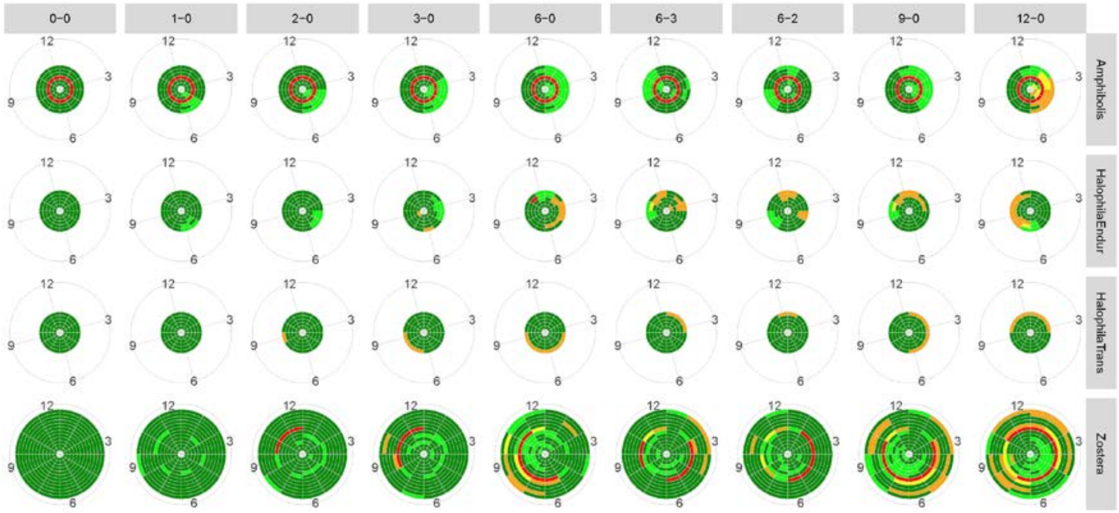

Supplementary Fig. 15 Growth and Senescent Season Effects on Recovery Time

Realised shoot density for dredging in September of duration 6 months (top) and 12 months (bottom) showing realised shoot density (left) and seed density (right). Although dredging drives the population to zero in both cases, there are sufficient surviving seeds to enable recovery although there is higher probability of high seed density for 6 months dredging compared to 12 months. However, as 12 months dredging completes at the beginning of the growth season (bottom), recovery begins right away whereas there is a 6 month delay for dredging of 6 months duration (top).

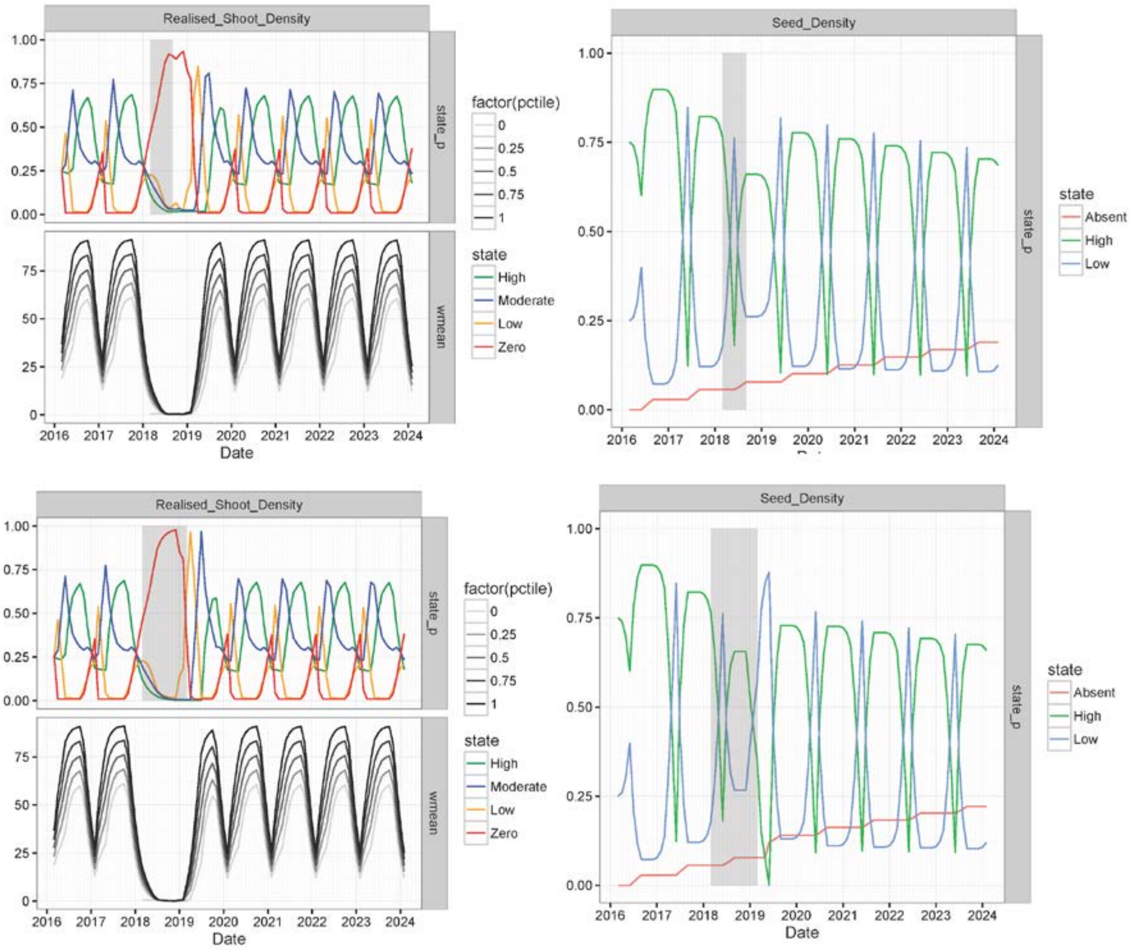

Supplementary Table 1. Improvement Statistics

This table gives the mean and standard deviation (sd) in the ratio of recovery time and extinction risk between the best start time for dredging (often corresponding to an ecological window) and the worst start time for 0% light during dredging. A ratio of 0.25 for recovery time for instance describes a fourfold improvement. These are aggregated over dredging durations ranging from 1 month to 3 months in part 1; part 2 also includes 6 months of dredging with alternating 2 months or 3 months of rest (6-2 and 6-3 respectively). Note that a ratio of zero implies the optimal window results in a recovery time of zero months (i.e. no impact).

	**Part 1: Up to 3 months dredging**				**Part 2: Up to 3 months dredging and 6-2 and 6-3**			
**Genera**	Mean Ratio of Recovery Time	SD in Ratio of Recovery Time	Mean Ratio of Extinction Risk	SD in Ratio of Extinction Risk	Mean Ratio of Recovery Time	SD in Ratio of Recovery Time	Mean Ratio of Extinction Risk	SD in Ratio of Extinction Risk
Amphibolis	0.6	0.42	0.83	0.1	0.67	0.35	0.83	0.1
**Enduring *Halophila***	0.23	0.09	0.88	0.04	0.28	0.12	0.88	0.04
**Transitory *Halophila***	0	0	0.84	0.05	0.25	0.33	0.84	0.08
Zostera	0.18	0.23	0.71	0.13	0.28	0.25	0.71	0.13

Supplementary Table 2. Global Scenarios

Inputs to the model covering genera, light saturation probability by month of year, the meadow type, habitat, contact person for each site and reference papers for the site and for the light saturation threshold used. Contact person: GK = Gary Kendrick, KM = Kathryn McMahon, MR = Michael Rasheed, KC = Katie Chartrand, MN = Masahiro Nakaoka, GW = Gidon Winters, DB = David Ball, DK-J = Dorte Krause-Jensen, JG = Jeffrey Gaeckle. Meadow type: Trans = Transitory, Endur = Enduring. Habitat: DST = Deep SubTidal, SST = Shallow SubTidal, IT = InterTidal. NP = Data not yet published.

Port	Genera	Jan	Feb	Mar	Apr	May	Jun	Jul	Aug	Sep	Oct	Nov	Dec	Meadow Type	Tropical-Temperate	Habitat	Latitude	Longitude	Contact Person	Ref	Saturation threshold reference
**James Price Point, Australia**	Halophila	0.38	0.38	0.38	0.38	0.77	0.77	0.77	0.77	0.77	0.77	0.38	0.38	Trans	Tropical	DST	-17.5156	122.1298	GK	2	3
**Jurien Bay, Australia**	Amphibolis	1	1	0.89	0.77	1	1	1	1	1	1	1	1	Endur	Temperate	SST	-30.2818	114.9717	KM	4	4
**Singapore**	Halophila	0.75	0.75	0.75	1	1	1	1	1	1	0.75	0.75	0.75	Endur	Tropical	IT	1.315402	103.5668	KM	5	5
**Mombasa, Kenya**	Halophila	1	1	0.75	0.75	0.75	0.75	1	1	1	1	0.8	0.8	Endur	Tropical	IT	-4.03501	39.59615	KM	6	7
**Hay Point, Australia**	Halophila	0.26	0.11	0.10	0.20	0.23	0.27	0.50	0.61	0.60	0.59	0.50	0.39	Trans	Tropical	DST	-21.2978	149.2346	MR	8	9
**Hay Point Inshore, Australia**	Halophila	0.4	0.2	0.2	0.2	0.2	0.4	0.5	0.7	0.7	0.7	0.7	0.5	Trans	Tropical	DST	-21.2978	149.2346	MR	8	9
**Adelaide Waters, Australia**	Amphibolis	1	1	1	1	1	0.5	0.5	0.5	0.5	0.5	0.5	0.5	Endur	Temperate	SST	-34.8082	138.5043	KM	10	11
**Adelaide Waters Site 2, Australia**	Amphibolis	1	1	1	1	1	1	1	0.75	1	0.9	0.75	0.5	Endur	Temperate	SST	-34.8082	138.5043	KM	10	11
**Cockburn Sound, Australia**	Amphibolis	1	1	1	1	1	1	1	1	1	1	1	1	Endur	Temperate	SST	-32.2059	115.7083	KM	12	12
**Geraldton, Australia**	Amphibolis	1	1	1	1	1	0.89	0.77	1	1	1	1	1	Endur	Temperate	SST	-28.7764	114.6052	KM	13	11
**Gladstone, Australia**	Zostera	0.66	0.55	0.58	0.87	0.47	0.47	0.82	0.90	0.92	0.97	0.95	0.91	Endur	Tropical	IT	-23.8244	151.2704	KC	14	15
**Aininkap, Japan**	Zostera	0.4	1	1	1	1	1	1	1	1	1	0.4	0.4	Endur	Temperate	SST	43.02	144.84	MN & KM	16	7
**Moreton Bay (western side Waterloo Bay),**	Zostera	0.83	0.67	0.88	0.38	0.5	0.88	0.5	0.88	0.88	0.75	0.75	1	Endur	Tropical	IT	-27.2725	153.1234	KM	17	17

**Australia**																					
**Red Sea Phosphate Terminal, Israel**	Halophila	1.00	0.99	0.78	0.87	0.64	1	1	1	1	0.88	0.88	0.98	Endur	Tropical	DST	29.4873	34.90513	GW	NP	[18]
**Red Sea Open Sea, Israel**	Halophila	1.00	0.99	0.98	0.91	0.66	1	1	1	1	1	1	0.98	Endur	Tropical	DST	29.4873	34.90513	GW	NP	[18]
**Red Sea Navy, Israel**	Halophila	0.73	0.62	1.00	0.73	0.62	1.00	1	1	1.00	0.87	1	0.98	Endur	Tropical	DST	29.4873	34.90513	GW	NP	[18]
**Point Franklin, Port of Melbourne, Australia**	Amphibolis	1	1	1	1	1	1	1	1	1	1	1	1	Endur	Temperate	SST	-38.2729	144.7682	DB	[19]	[19]
**Mud Island, Port of Melbourne, Australia**	Zostera	1	1	1	1	1	0.7	0.4	0.5	0.9	1	1	1	Endur	Temperate	SST	-38.2729	144.7682	DB	[19]	[19]
**Cameron's Bight, Port of Melbourne, Australia**	Zostera	1	1	1	0.7	0.4	0.2	0.3	0.3	0.4	0.7	1	1	Endur	Temperate	SST	-38.2729	144.7682	DB	[19]	[19]
**Kobbefjord, Greenland**	Zostera	0	0	1	0.93	0.81	1	0.94	0.94	0.77	0.84	0	0	Trans	Temperate	SST	64.14683	-51.6041	DK-J	[20]	[21]
**Ameralik, Greenland**	Zostera	0	0	0	0.83	0.97	0.9	1	0.88	0.89	0	0	0	Trans	Temperate	SST	64.14683	-51.6041	DK-J	[20]	[21]
**Kapisillit, Greenland**	Zostera	0	0	0	0	0.77	0.77	0.74	0.79	0.90	0	0	0	Trans	Temperate	SST	64.14683	-51.6041		[20]	[21]
**Roskilde, Denmark**	Zostera	0.95	0.9	0.95	0.9	0.95	0.85	0.8	0.6	0.6	0.75	0.85	0.8	Endur	Temperate	SST	55.67026	11.94122	KM	[22]	[22]
**Waquoit Bay, USA**	Zostera	0.75	1	1	1	1	1	1	1	0.5	0.75	1	1	Endur	Temperate	SST	41.56576	-70.5427	KM	[23]	[21]
**Abbot Point, Australia**	Halophila	0.60	0.39	0.23	0.08	0.06	0.12	0.4	0.46	0.82	0.56	0.64	0.52	Trans	Tropical	DST	-19.8971	148.0873	KC	[8]	[9]
**Puget Sound A**	Zostera	0.00	0.02	0.63	0.95	0.99	1.00	1.00	0.53	0.04	0.00	0.00	0.00	Endur	Temperate	SST	47.32862	-122.391	JG	NP	[24]
**Salt River Canyon**	Halophila	0.86	0.92	1	1	1	1	1	0.94	0.65	0.75	0.71	0.67	Trans	Tropical	DST	17.77677	-64.7552	KM	[25]	[25]
**Puget Sound C**	Zostera	0.021	0.268	0.933	0.995	0.998	1	0.998	0.981	0.505	0.051	0.008	0.008	Endur	Temperate	SST	47.32946	-122.391	JG	NP	[7]

Supplementary Table 3. Node Definitions

For the Dynamics Bayesian Network (DBN) shown in Supplementary Fig. 7 – 11, a definition of the nodes is provided below. Note that the model is evaluated on a monthly time scale in line with seagrass growth dynamics[26].

Node	Definition	States
**Realised Shoot Density**	Number of shoots, clusters of leaves or leaf pairs per m^2	High, moderate, low, zero (see Method for discussion on thresholds)
**Realised Biomass**	Grams of dry matter per m^2	High, moderate, low, zero (see Method for discussion on thresholds)
**Aerial Extent**	Meadow area in m^2	Increase or decrease
**Time of Year**	Month of the year	January through December
**Genera Presence**	Categorical, proportion of meadow of that genera	*Halophila, Amphibolis, Enhalus, Thalassia, Cymodocea, Halodule, Syringodium, Thalassodendron, Posidonia, Phyllospadix, Zostera, Ruppia, Lepilaena*
**Habitat**	Categorical, relating to location of the meadow in the coast	InterTidal

		ShallowSubTidal<br>DeepSubTidal
**Tropical-Temperate**	Categorical, whether the meadow is located in tropical or temperate climates	Tropical<br>Temperate
**Meadow Type**	Categorical, whether the meadow is present year-round or not [27]	Transitory<br>Enduring
**Location Type**	An aggregate of habitat, tropical-temperate and meadow type	All combinations of states from habitat, tropical-temperate, and meadow type
**Seed Density**	Density of seeds per m^2. States capture the dynamic range in growth rates from fast colonising species to slow persistent species [28]	High, low, absent
**Recruitment Rate from Seeds**	Rate of recruitment into the adult population from seeds	High, low, absent
**Seed Quality**	Quality score of the seed that affects successful recruitment rate	High, low
**Immigrant Seed Density**	Capture connectivity between meadows with immigrant seed density	High, low, absent
**Immigrant Seed**	Capture connectivity between meadows with immigrant seed quality	High, low

**Quality**		
**Lateral Growth from Existing Individuals**	Rate of lateral growth of rhizomes representing all existing individuals in a meadow. States capture the dynamic range in growth rates from fast colonising species to slow persistent species [28]	Fast, moderate, slow, zero
**Overall Lateral Growth**	Combined lateral growth rate from existing and immigrant individuals	Fast, moderate, slow, zero
**Immigrant Vegetative Fragments**	Capture connectivity between meadows with immigrant vegetative fragments	High, low, zero
**Ability to Recover**	Aggregated score of the ability for the meadow to recover	High, low, zero
**Rate of Recovery in Shoot Density**	Rate of recovery in shoot density in that month	High, moderate, low, zero
**Rate of Recovery in Biomass**	Rate of recovery in biomass in that month	High, moderate, low, zero
**Physiological status of plants**	The physiological status captures the degree to which the plant is able to function normally. It can be approximated by but is not wholly	Good, medium, poor

	represented by rhizome carbohydrate stores where good represent build-up of stores and normal function, medium is using reserves and functioning normally and poor is unable to function normally.	
**Above to below ground biomass ratio**	Represents the ratio of above to below ground biomass where large amounts of below ground biomass suggest stronger resistance to stress	More above, more below
**Ability to resist hazard**	Aggregate score similar to ability to recover but focused on ability to resist a hazard (i.e. not to suffer a loss)	Strong, weak
**Accumulated light**	Probability of meeting light requirements for normal function of the plant representing accumulated variations and effects in that month. This is not the same as benthic light as it must take into account the light requirements of that specific plant at that location.	Above saturation, below saturation
**Accumulated burial**	Probability of experiencing an effect accumulated over a month from burial by sediment, taking into account the particular plant and location.	Effect, no effect
**Sediment Quality**	Probability of experience an effect accumulated over a month from	Effect, no effect

	sediment quality such as exposure to sulphites compromising photosynthetic process, taking into account the particular plant and location.	
**Temperature**	The suitability, temperature wise to optimal plant function for that plant, location	Optimal, sub-optimal
**Salinity**	The suitability, salinity wise to optimal plant function for that plant, location	Optimal, sub-optimal
**Loss in Shoot Density**	Loss in shoot density for that month	High, moderate, low, zero
**Loss in Biomass**	Loss in biomass for that month	High, moderate, low, zero
**Baseline Shoot Density**	Best case expected shoot density for a given month given the physiological status of the meadow. Used to explicitly capture large seasonal variations.	High, moderate, low, zero
**Baseline Biomass**	Best case expected biomass for a given month given the physiological status of the meadow. Used to explicitly capture large seasonal variations.	High, moderate, low, zero

**Net Change Shoot Density**	Probabilistic subtraction of loss in shoot density from realised shoot density then adding rate of recovery in shoot density	High, moderate, low, zero
**Net Change Biomass**	Probabilistic subtraction of loss in biomass from realised biomass then adding rate of recovery in biomass	High, moderate, low, zero

Supplementary Table 4. Expert Validation

Count of experts and the level of confidence they expressed about different aspects of the model. Input factors relate to nodes coloured white in Supplementary Fig. 7, recovery factors to Supplementary Fig. 8, resistance factors to Supplementary Fig. 9, and population (shoot density and biomass) coloured yellow in Supplementary Fig. 7 through 9 and 11[29].

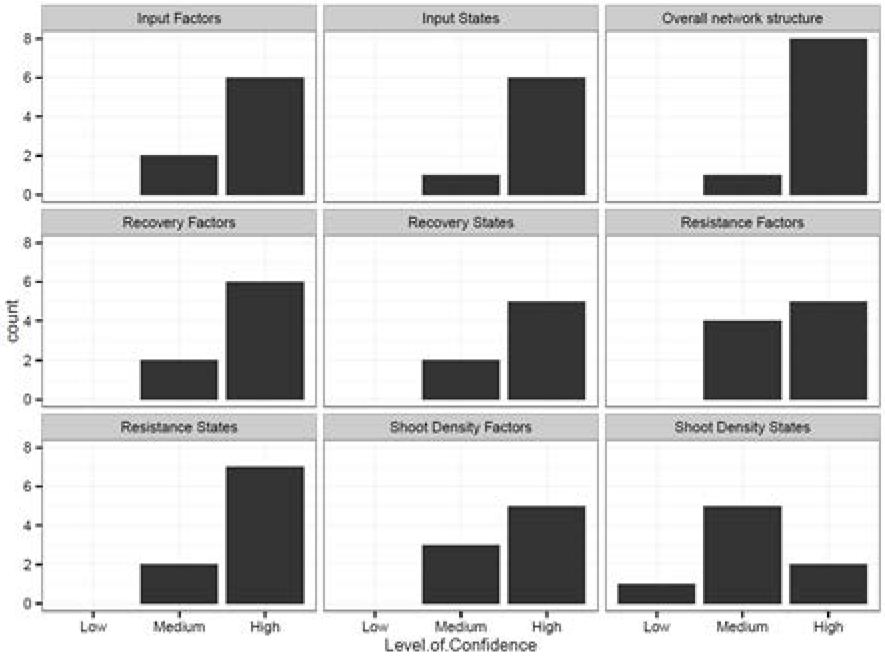

Supplementary Table 5. Expert Panel

Expert involvement in elicitation and validation along with their area of expertise and a link to their Google Scholar profile if available.

Expert (with Google Scholar link)	Network Development	Network Validation	Scenario Validation	Category of Expert	Expertise
Kathryn McMahon	Yes	Yes	Yes	Specialist	Seagrass
Gary Kendrick	Yes	Yes	Yes	Specialist	Seagrass
Paul Lavery	Yes	No	Yes	Specialist	Seagrass
Julian Caley	Yes	No	Yes	General	Marine Ecology
Michael Rasheed	Yes	No	Yes	Specialist	Seagrass
Paul York	Yes	No	Yes	Specialist	Seagrass
Katie Chartrand	Yes	No	Yes	Specialist	Seagrass
John Huisman	No	Yes	No	General	Algae
Andy Davis	No	Yes	No	General	Invertebrates
Jessie Short	No	Yes	No	General	Coral
Ashley Lemmon	No	Yes	No	General	Environmental monitoring for dredging
John Keesing	No	Yes	No	General	Marine Ecology
Dianne McLean	No	Yes	No	General	Fish
Paul Erftemeijer	No	Yes	No	Specialist	Seagrass

Supplementary Table 6. ***Amphibolis*** **Validation Light**

Probability of above saturation light for control, moderate and high shading designs used as input for model validation of *Amphibolis* at Jurien Bay, Australia[4,11].

Month	Control	Moderate	High
**1**	0.998186	0.221183	0.142531
**2**	0.998043	0.278875	0.005148
**3**	0.998802	0.208029	0.030681
**4**	0.984657	0.115154	0.014997
**5**	0.890455	0.004855	0.003143
**6**	0.771024	0.005142	0.00332
**7**	0.998267	0.007945	0.004558
**8**	0.998201	0.008472	0.004739
**9**	0.99811	0.008805	0.005023
**10**	0.998227	0.698312	0.004749
**11**	0.998036	0.456991	0.004994

**12**	0.998134	0.379656	0.053117

Supplementary Table 7. *Amphibolis* Validation Responses

Data derived state probabilities for shoot density, biomass, lateral growth and physiological status corresponding to the experimental designs and light scenarios (Supplementary Table 6) used for validation of *Amphibolis* at Jurien Bay, Australia[4,11].

					Shoot Density				Biomass				Lateral Growth				Physiological Status		
	Timing	Duration	Intensity	Date	Zero	Low	Moderate	High	Zero	Low	Moderate	High	Zero	Slow	Moderate	Fast	Poor	Medium	Good
1	Summer	0	Control	9/03/2005	0.10	0.02	0.26	0.62	0.01	0.05	0.41	0.54	0.02	0.98	0.00	0.00	0.02	0.02	0.96
2	Summer	3	Control	13/06/2005	0.06	0.05	0.61	0.27	0.04	0.06	0.39	0.51	0.07	0.93	0.00	0.00	0.12	0.12	0.76
3	Summer	3	Moderate	13/06/2005	0.02	0.01	0.83	0.13	0.03	0.04	0.81	0.12	0.59	0.41	0.00	0.00	0.36	0.41	0.23
4	Summer	3	High	13/06/2005	0.03	0.11	0.86	0.01	0.01	0.07	0.90	0.02	0.84	0.16	0.00	0.00	0.43	0.41	0.17
5	Summer	6	Control	15/09/2005	0.17	0.13	0.49	0.21	0.06	0.16	0.60	0.18	0.05	0.95	0.00	0.00	0.38	0.35	0.27
6	Summer	6	Moderate	15/09/2005	0.07	0.07	0.81	0.04	0.10	0.28	0.58	0.04	1.00	0.00	0.00	0.00	0.46	0.42	0.12

**7**	Summer	6	High	15/09/2005	0.17	0.13	0.49	0.21	0.06	0.16	0.60	0.18	1.00	0.00	0.00	0.00	0.38	0.35	0.27
**8**	Summer	9	Control	28/11/2005	0.36	0.10	0.25	0.29	0.02	0.17	0.57	0.24	0.02	0.98	0.00	0.00	0.13	0.14	0.73
**9**	Summer	9	Moderate	28/11/2005	0.36	0.10	0.25	0.29	0.02	0.17	0.57	0.24	1.00	0.00	0.00	0.00	0.13	0.14	0.73
**10**	Summer	9	High	28/11/2005	0.99	0.01	0.00	0.00	0.05	0.35	0.52	0.08	0.49	0.51	0.00	0.00	0.94	0.06	0.00
**11**	Summer	3	Control	6/10/2005	0.08	0.04	0.44	0.45	0.01	0.05	0.40	0.53	0.03	0.97	0.00	0.00	0.05	0.05	0.91
**12**	Summer	3	Moderate	6/10/2005	0.04	0.01	0.68	0.27	0.01	0.04	0.79	0.16	0.30	0.70	0.00	0.00	0.20	0.24	0.56
**13**	Summer	3	High	6/10/2005	0.05	0.09	0.84	0.02	0.00	0.06	0.90	0.03	0.56	0.44	0.00	0.00	0.26	0.27	0.48
**14**	Summer	6	Control	29/08/2007	0.15	0.14	0.54	0.17	0.09	0.16	0.57	0.17	0.06	0.94	0.00	0.00	0.42	0.37	0.21
**15**	Summer	6	Moderate	29/08/2007	0.06	0.07	0.83	0.03	0.14	0.28	0.55	0.04	0.06	0.94	0.00	0.00	0.48	0.43	0.09
**16**	Summer	6	High	29/08/2007	0.15	0.14	0.54	0.17	0.09	0.16	0.57	0.17	0.06	0.94	0.00	0.00	0.42	0.37	0.21
**17**	Summer	3	Control	5/11/2006	0.09	0.03	0.32	0.56	0.01	0.05	0.41	0.53	0.02	0.98	0.00	0.00	0.02	0.03	0.95

18	Summer	3	Moderate	5/11/2006	0.07	0.02	0.40	0.51	0.01	0.04	0.56	0.39	0.05	0.95	0.00	0.00	0.04	0.05	0.91
19	Summer	3	High	5/11/2006	0.11	0.05	0.58	0.26	0.01	0.06	0.67	0.26	0.06	0.94	0.00	0.00	0.05	0.05	0.90
20	Winter	0	Control	15/09/2005	0.07	0.04	0.52	0.37	0.02	0.06	0.40	0.52	0.05	0.95	0.00	0.00	0.07	0.07	0.86
21	Winter	3	Control	19/12/2005	0.11	0.02	0.19	0.69	0.00	0.04	0.41	0.54	0.01	0.99	0.00	0.00	0.01	0.01	0.98
22	Winter	3	Moderate	19/12/2005	0.06	0.01	0.43	0.51	0.00	0.03	0.85	0.12	0.20	0.80	0.00	0.00	0.08	0.12	0.80
23	Winter	3	High	19/12/2005	0.12	0.09	0.76	0.03	0.00	0.05	0.93	0.02	0.47	0.53	0.00	0.00	0.12	0.15	0.73
24	Winter	6	Control	28/03/2006	0.24	0.09	0.33	0.34	0.02	0.14	0.64	0.19	0.02	0.98	0.00	0.00	0.23	0.24	0.53
25	Winter	6	Moderate	28/03/2006	0.13	0.06	0.72	0.09	0.04	0.26	0.65	0.05	1.00	0.00	0.00	0.00	0.36	0.37	0.27
26	Winter	6	High	28/03/2006	0.24	0.09	0.33	0.34	0.02	0.14	0.64	0.19	1.00	0.00	0.00	0.00	0.23	0.24	0.53
27	Winter	9	Control	3/07/2006	0.14	0.22	0.56	0.08	0.20	0.19	0.43	0.18	0.10	0.90	0.00	0.00	0.46	0.37	0.17
28	Winter	9	Moderate	3/07/2006	0.14	0.22	0.56	0.08	0.20	0.19	0.43	0.18	1.00	0.00	0.00	0.00	0.46	0.37	0.17

29	Winter	9	High	3/07/2006	0.95	0.05	0.00	0.00	0.35	0.31	0.30	0.04	0.49	0.51	0.00	0.00	0.95	0.05	0.00
30	Winter	3	Control	27/03/2006	0.09	0.03	0.32	0.56	0.01	0.05	0.41	0.53	0.02	0.98	0.00	0.00	0.02	0.03	0.95
31	Winter	3	Moderate	27/03/2006	0.05	0.01	0.57	0.37	0.01	0.03	0.81	0.15	0.23	0.77	0.00	0.00	0.14	0.17	0.69
32	Winter	3	High	27/03/2006	0.08	0.09	0.81	0.03	0.00	0.05	0.91	0.03	0.50	0.50	0.00	0.00	0.19	0.21	0.61
33	Winter	3	Control	5/11/2006	0.09	0.03	0.32	0.56	0.01	0.05	0.41	0.53	0.02	0.98	0.00	0.00	0.02	0.03	0.95
34	Winter	3	Moderate	5/11/2006	0.06	0.01	0.49	0.44	0.01	0.04	0.71	0.25	0.11	0.89	0.00	0.00	0.08	0.09	0.83
35	Winter	3	High	5/11/2006	0.10	0.07	0.75	0.09	0.00	0.06	0.85	0.09	0.20	0.80	0.00	0.00	0.10	0.11	0.79
36	Winter	6	Control	29/08/2007	0.15	0.14	0.54	0.17	0.09	0.16	0.57	0.17	0.06	0.94	0.00	0.00	0.42	0.37	0.21
37	Winter	6	Moderate	29/08/2007	0.06	0.07	0.83	0.03	0.14	0.28	0.55	0.04	0.98	0.02	0.00	0.00	0.48	0.43	0.09
38	Winter	6	High	29/08/2007	0.15	0.14	0.54	0.17	0.09	0.16	0.57	0.17	0.98	0.02	0.00	0.00	0.42	0.37	0.21
39	Winter	9	Control	29/08/2007	0.19	0.19	0.49	0.12	0.11	0.19	0.49	0.20	0.06	0.94	0.00	0.00	0.39	0.33	0.28

**40**	Winter	9	Moderate	29/08/2007	0.19	0.19	0.49	0.12	0.11	0.19	0.49	0.20	0.99	0.01	0.00	0.00	0.39	0.33	0.28
**41**	Winter	9	High	29/08/2007	0.85	0.15	0.00	0.00	0.22	0.35	0.38	0.05	0.48	0.52	0.00	0.00	0.82	0.18	0.00

Supplementary Table 8. Amphibolis MSE

Mean Squared Error (MSE) in predicted state probabilities for shoot density, biomass, lateral growth and physiological status corresponding to the experimental designs and light scenarios (Supplementary Table 6 – 7) used for validation of *Amphibolis* at Jurien Bay, Australia[4,11].

Timing	Duration	Intensity	Shoot Density	Biomass	Lateral Growth	Physiological Status
**Summer**	0	Control	0.024003	0.017324	0.00466	0.042158
**Summer**	3	Moderate	0.018761	0.018767	0.005911	0.037642
**Summer**	3	High	0.024663	0.03145	0.046276	0.051881
**Summer**	6	Moderate	0.039727	0.039293	0.015209	*NA*
**Summer**	6	High	0.020848	0.034172	0.014355	*NA*
**Summer**	9	Moderate	0.013544	0.06675	*NA*	*NA*
**Summer**	9	High	0.016583	0.101594	*NA*	*NA*
**Winter**	0	Control	0.027037	0.019832	0.002731	0.062617
**Winter**	3	Moderate	0.002893	0.034127	0.003727	0.025627
**Winter**	3	High	0.027626	0.045085	0.011673	0.089454

**Winter**	6	Moderate	0.017472	0.032982	0.049432	*NA*
**Winter**	6	High	0.015767	0.030588	0.018043	*NA*
**Winter**	9	Moderate	0.072291	0.06482	0.006441	*NA*
**Winter**	9	High	0.008118	0.059785	*NA*	*NA*

Supplementary Table 9. *Amphibolis* Zero State MSE

Mean Squared Error (MSE) in predicted probability for the zero state probabilities for shoot density, biomass and lateral growth corresponding to the experimental designs and light scenarios (Supplementary Table 6 – 7) used for validation of *Amphibolis* at Jurien Bay, Australia[4,11].

	Timing	Duration	Intensity	Shoot Density	Biomass	Lateral Growth	Physiological Status
1	Summer	0	Control	0.023985	0.000222	0.007451	NA
3	Summer	3	Moderate	0.004501	0.000618	0.012758	NA
4	Summer	3	High	0.006347	0.001417	0.096682	NA
6	Summer	6	Moderate	0.043897	0.039506	0.031398	NA
7	Summer	6	High	0.032571	0.048011	0.029608	NA
9	Summer	9	Moderate	0.023403	0.141713	NA	NA
10	Summer	9	High	0.035775	0.238599	NA	NA
20	Winter	0	Control	0.014357	0.003237	0.004027	NA
22	Winter	3	Moderate	0.002198	0.000799	0.006561	NA
23	Winter	3	High	0.004218	0.003141	0.024289	NA
2	Winter	6	Moderate	0.002593	0.00979	0.101608	NA

**5**							
**2 6**	Winter	6	High	0.015143	0.046075	0.036911	*NA*
**2 8**	Winter	9	Moderate	0.09922	0.072914	0.012503	*NA*
**2 9**	Winter	9	High	0.019135	0.148147	*NA*	*NA*

Supplementary Table 10. *Halophila* Validation Responses

Data derived state probabilities for biomass for four sites before and after dredging used for validation of *Halophila* at Hay Point, Australia[8].

	Date	Location	DredgeCategory	Zero	Low	Moderate	High
1	1/07/2004	Control Inshore	PreDredge	0.7520	0.1309	0.1120	0.0050
2	1/07/2004	Control Offshore	PreDredge	0.7520	0.1309	0.1120	0.0050
3	1/07/2004	Dredge Plume	PreDredge	0.7520	0.1309	0.1120	0.0050
4	1/07/2004	Spoil Ground	PreDredge	0.7520	0.1309	0.1120	0.0050
5	1/12/2005	Control Inshore	PreDredge	0.9603	0.0055	0.0018	0.0325
6	1/12/2005	Control Offshore	PreDredge	0.9603	0.0055	0.0018	0.0325
7	1/12/2005	Dredge Plume	PreDredge	0.9603	0.0055	0.0018	0.0325
8	1/12/2005	Spoil Ground	PreDredge	0.9603	0.0055	0.0018	0.0325
9	1/03/2006	Control Inshore	PreDredge	0.9611	0.0058	0.0019	0.0313
10	1/03/2006	Control Offshore	PreDredge	0.9611	0.0058	0.0019	0.0313
11	1/03/2006	Dredge Plume	PreDredge	0.9611	0.0058	0.0019	0.0313
12	1/03/2006	Spoil Ground	PreDredge	0.9611	0.0058	0.0019	0.0313
13	1/05/2006	Control Inshore	Dredge	0.9458	0.0283	0.0142	0.0117
14	1/05/2006	Control Offshore	Dredge	0.9971	0.0027	0.0002	0.0000
15	1/05/2006	Dredge Plume	Dredge	0.9995	0.0003	0.0002	0.0000
16	1/05/2006	Spoil Ground	Dredge	0.9995	0.0003	0.0002	0.0000
17	1/07/2006	Control Inshore	Dredge	0.7520	0.1309	0.1120	0.0050

18	1/07/2006	Control Offshore	Dredge	0.9825	0.0156	0.0019	0.0000
19	1/07/2006	Dredge Plume	Dredge	0.9962	0.0019	0.0019	0.0000
20	1/07/2006	Spoil Ground	Dredge	0.9959	0.0020	0.0021	0.0000
21	1/08/2006	Control Inshore	Dredge	0.7642	0.1254	0.1052	0.0052
22	1/08/2006	Control Offshore	Dredge	0.9835	0.0147	0.0018	0.0000
23	1/08/2006	Dredge Plume	Dredge	0.9964	0.0018	0.0018	0.0000
24	1/08/2006	Spoil Ground	Dredge	0.9962	0.0019	0.0019	0.0000
25	1/09/2006	Control Inshore	Dredge	0.8954	0.0591	0.0374	0.0080
26	1/09/2006	Control Offshore	Dredge	0.9935	0.0060	0.0005	0.0000
27	1/09/2006	Dredge Plume	Dredge	0.9988	0.0007	0.0005	0.0000
28	1/09/2006	Spoil Ground	Dredge	0.9987	0.0007	0.0006	0.0000
29	1/10/2006	Control Inshore	Dredge	0.9478	0.0268	0.0133	0.0121
30	1/10/2006	Control Offshore	Dredge	0.9972	0.0026	0.0002	0.0000
31	1/10/2006	Dredge Plume	Dredge	0.9995	0.0003	0.0002	0.0000
32	1/10/2006	Spoil Ground	Dredge	0.9995	0.0003	0.0002	0.0000
33	1/11/2006	Control Inshore	Dredge	0.9642	0.0118	0.0047	0.0193
34	1/11/2006	Control Offshore	Dredge	0.9988	0.0011	0.0001	0.0000
35	1/11/2006	Dredge Plume	Dredge	0.9998	0.0001	0.0001	0.0000
36	1/11/2006	Spoil Ground	Dredge	0.9998	0.0001	0.0001	0.0000
37	1/01/2007	Control Inshore	Dredge	0.9374	0.0025	0.0007	0.0594
38	1/01/2007	Control Offshore	Dredge	0.9997	0.0002	0.0000	0.0001
39	1/01/2007	Dredge Plume	Dredge	0.9999	0.0000	0.0000	0.0000

40	1/01/2007	Spoil Ground	Dredge	0.9999	0.0000	0.0000	0.0000
41	1/03/2007	Control Inshore	PostDredge	0.9611	0.0058	0.0019	0.0313
42	1/03/2007	Control Offshore	PostDredge	0.9992	0.0007	0.0000	0.0000
43	1/03/2007	Dredge Plume	PostDredge	0.9998	0.0001	0.0000	0.0000
44	1/03/2007	Spoil Ground	PostDredge	0.9998	0.0001	0.0000	0.0000
45	1/07/2007	Control Inshore	PostDredge	0.7520	0.1309	0.1120	0.0050
46	1/07/2007	Control Offshore	PostDredge	0.9745	0.0220	0.0034	0.0000
47	1/07/2007	Dredge Plume	PostDredge	0.9929	0.0036	0.0035	0.0000
48	1/07/2007	Spoil Ground	PostDredge	0.9926	0.0037	0.0037	0.0000
49	1/09/2007	Control Inshore	PostDredge	0.8954	0.0591	0.0374	0.0080
50	1/09/2007	Control Offshore	PostDredge	0.9900	0.0089	0.0010	0.0000
51	1/09/2007	Dredge Plume	PostDredge	0.9974	0.0015	0.0011	0.0000
52	1/09/2007	Spoil Ground	PostDredge	0.9973	0.0015	0.0011	0.0000
53	1/11/2007	Control Inshore	PostDredge	0.9642	0.0118	0.0047	0.0193
54	1/11/2007	Control Offshore	PostDredge	0.9981	0.0018	0.0001	0.0000
55	1/11/2007	Dredge Plume	PostDredge	0.9996	0.0003	0.0001	0.0000
56	1/11/2007	Spoil Ground	PostDredge	0.9995	0.0003	0.0001	0.0000
57	1/02/2008	Control Inshore	PostDredge	0.9426	0.0028	0.0008	0.0537
58	1/02/2008	Control Offshore	PostDredge	0.9994	0.0005	0.0000	0.0001
59	1/02/2008	Dredge Plume	PostDredge	0.9998	0.0001	0.0000	0.0001
60	1/02/2008	Spoil Ground	PostDredge	0.9998	0.0001	0.0000	0.0001
61	1/06/2008	Control Inshore	PostDredge	0.8839	0.0656	0.0429	0.0076

62	1/06/2008	Control Offshore	PostDredge	0.9853	0.0128	0.0018	0.0000
63	1/06/2008	Dredge Plume	PostDredge	0.9955	0.0026	0.0019	0.0000
64	1/06/2008	Spoil Ground	PostDredge	0.9954	0.0027	0.0020	0.0000
65	1/06/2009	Control Inshore	PostDredge	0.8839	0.0656	0.0429	0.0076
66	1/06/2009	Control Offshore	PostDredge	0.9791	0.0176	0.0032	0.0000
67	1/06/2009	Dredge Plume	PostDredge	0.9920	0.0047	0.0033	0.0000
68	1/06/2009	Spoil Ground	PostDredge	0.9918	0.0048	0.0034	0.0000
69	1/10/2009	Control Inshore	PostDredge	0.9488	0.0261	0.0128	0.0123
70	1/10/2009	Control Offshore	PostDredge	0.9914	0.0075	0.0011	0.0000
71	1/10/2009	Dredge Plume	PostDredge	0.9967	0.0022	0.0011	0.0000
72	1/10/2009	Spoil Ground	PostDredge	0.9966	0.0022	0.0012	0.0000
73	1/11/2010	Control Inshore	PostDredge	0.9643	0.0115	0.0045	0.0196
74	1/11/2010	Control Offshore	PostDredge	0.9940	0.0050	0.0008	0.0001
75	1/11/2010	Dredge Plume	PostDredge	0.9970	0.0021	0.0008	0.0001
76	1/11/2010	Spoil Ground	PostDredge	0.9970	0.0021	0.0009	0.0001
77	1/04/2011	Control Inshore	PostDredge	0.9634	0.0131	0.0053	0.0181
78	1/04/2011	Control Offshore	PostDredge	0.9917	0.0068	0.0013	0.0001
79	1/04/2011	Dredge Plume	PostDredge	0.9953	0.0033	0.0014	0.0001
80	1/04/2011	Spoil Ground	PostDredge	0.9952	0.0033	0.0014	0.0001
81	1/10/2011	Control Inshore	PostDredge	0.9488	0.0261	0.0128	0.0123
82	1/10/2011	Control Offshore	PostDredge	0.9780	0.0169	0.0049	0.0002
83	1/10/2011	Dredge Plume	PostDredge	0.9848	0.0101	0.0050	0.0001

**84**	1/10/2011	Spoil Ground	PostDredge	0.9847	0.0101	0.0050	0.0001
**85**	1/04/2012	Control Inshore	PostDredge	0.9632	0.0135	0.0055	0.0178
**86**	1/04/2012	Control Offshore	PostDredge	0.9853	0.0109	0.0033	0.0005
**87**	1/04/2012	Dredge Plume	PostDredge	0.9881	0.0081	0.0033	0.0004
**88**	1/04/2012	Spoil Ground	PostDredge	0.9881	0.0082	0.0033	0.0005
**89**	1/10/2012	Control Inshore	PostDredge	0.9497	0.0254	0.0124	0.0124
**90**	1/10/2012	Control Offshore	PostDredge	0.9497	0.0254	0.0124	0.0124
**91**	1/10/2012	Dredge Plume	PostDredge	0.9497	0.0254	0.0124	0.0124
**92**	1/10/2012	Spoil Ground	PostDredge	0.9497	0.0254	0.0124	0.0124

Supplementary Table 11. *Halophila* Validation Salt River Canyon

Data derived state probabilities for biomass for four sites before and after dredging used for validation of *Halophila* at Salt River Canyon, St Croix, US Virgin Islands[25]. Data was simulated from the mean, variance and number of replicates reported in the paper assuming a normal distribution.

Date	Zero	Low	Moderate	High
**06/1983**	0.061981	0.154707	0.731642	0.05167
**07/1984**	0.110645	0.122423	0.686628	0.080303
**08/1984**	0.223172	0.172578	0.41198	0.19227
**11/1984**	0.926048	0.029069	0.015081	0.029803
**12/1984**	0.927112	0.029001	0.012961	0.030925
**01/1985**	0.916049	0.030536	0.01713	0.036285
**05/1985**	0.015914	0.065034	0.877993	0.041058
**07/1985**	0.15539	0.372786	0.413739	0.058085
**08/1985**	0.255609	0.12697	0.532799	0.084622
**10/1985**	0.201006	0.361235	0.381693	0.056067
**11/1985**	0.650361	0.182594	0.110715	0.05633
**12/1985**	0.921485	0.031585	0.01361	0.03332
**02/1986**	0.339791	0.495393	0.094447	0.070369
**03/1986**	0.041046	0.048411	0.831686	0.078857
**04/1986**	0.156949	0.200107	0.573019	0.069925

**05/1986**	0.221966	0.166389	0.509243	0.102402
**06/1986**	0.301794	0.207382	0.425189	0.065635
**07/1986**	0.116937	0.169537	0.515126	0.198399
**08/1986**	0.099015	0.125287	0.629995	0.145704
**09/1986**	0.125829	0.150824	0.672732	0.050614
**10/1986**	0.240516	0.406371	0.296603	0.05651
**11/1986**	0.922044	0.02986	0.0167	0.031396
**12/1986**	0.923445	0.029966	0.013476	0.033113

Supplementary Table 12. *Zostera* Validation Gladstone

Data derived state probabilities for light (probability of above saturation) and biomass corresponding to the experimental designs and light scenarios used for validation of *Zostera* at Gladstone, Australia[14].

Season	Treatment	Shading.Recovery	Date	light	Zero	Low	Moderate	High
**S1**	Medium	no shade	1/05/2010	0.90	0.02	0.04	0.77	0.16
**S1**	High	no shade	1/05/2010	0.87	0.03	0.05	0.63	0.29
**S1**	Low	no shade	1/05/2010	0.97	0.02	0.04	0.78	0.16
**S1**	Control	no shade	1/05/2010	0.97	0.02	0.04	0.78	0.16
**S1**	Medium	shading	1/06/2010	0.37	0.08	0.10	0.57	0.26
**S1**	High	shading	1/06/2010	0.27	0.10	0.11	0.40	0.39
**S1**	Low	shading	1/06/2010	0.53	0.05	0.07	0.73	0.15
**S1**	Control	shading	1/06/2010	0.87	0.03	0.05	0.63	0.29
**S1**	Medium	recovery	1/07/2010	0.94	0.02	0.05	0.64	0.29
**S1**	High	recovery	1/07/2010	0.94	0.02	0.05	0.48	0.45
**S1**	Low	recovery	1/07/2010	0.94	0.03	0.05	0.49	0.44
**S1**	Control	recovery	1/07/2010	0.94	0.03	0.05	0.50	0.42
**S1**	Medium	recovery	1/08/2010	1.00	0.02	0.04	0.78	0.16
**S1**	High	recovery	1/08/2010	1.00	0.02	0.04	0.50	0.44
**S1**	Low	recovery	1/08/2010	1.00	0.02	0.04	0.49	0.44

**S1**	Control	recovery	1/08/2010	1.00	0.02	0.04	0.35	0.58
**S1**	Medium	recovery	1/09/2010	0.91	0.02	0.05	0.64	0.29
**S1**	High	recovery	1/09/2010	0.91	0.03	0.05	0.64	0.29
**S1**	Low	recovery	1/09/2010	0.91	0.03	0.05	0.49	0.43
**S1**	Control	recovery	1/09/2010	0.91	0.02	0.05	0.34	0.58
**G1**	Low	no shade	1/09/2010	0.68	0.04	0.07	0.60	0.28
**G1**	Control	no shade	1/09/2010	0.95	0.02	0.05	0.64	0.29
**G1**	High	no shade	1/09/2010	0.00	0.14	0.13	0.50	0.23
**G1**	Medium	no shade	1/09/2010	0.36	0.08	0.10	0.28	0.55
**G1**	Low	shading	1/09/2010	0.68	0.04	0.07	0.46	0.43
**G1**	Control	shading	1/09/2010	0.95	0.02	0.04	0.20	0.73
**G1**	High	shading	1/09/2010	0.00	0.14	0.14	0.50	0.23
**G1**	Medium	shading	1/09/2010	0.36	0.08	0.10	0.27	0.55
**G1**	Low	shading	1/10/2010	0.65	0.05	0.07	0.47	0.41
**G1**	Control	shading	1/10/2010	0.87	0.03	0.05	0.33	0.59
**G1**	High	shading	1/10/2010	0.00	0.13	0.14	0.23	0.51
**G1**	Medium	shading	1/10/2010	0.39	0.07	0.09	0.16	0.69
**G1**	Low	shading	1/11/2010	0.23	0.08	0.10	0.70	0.13
**G1**	Control	shading	1/11/2010	0.80	0.03	0.06	0.48	0.43
**G1**	High	shading	1/11/2010	0.00	0.12	0.13	0.63	0.12
**G1**	Medium	shading	1/11/2010	0.13	0.12	0.12	0.39	0.37
**G1**	Low	shading	1/12/2010	0.65	0.05	0.53	0.22	0.20

**G1**	Control	shading	1/12/2010	0.68	0.04	0.07	0.46	0.43
**G1**	High	shading	1/12/2010	0.58	0.35	0.18	0.24	0.22
**G1**	Medium	shading	1/12/2010	0.58	0.06	0.54	0.21	0.19
**G1**	Low	recovery	1/01/2011	0.71	0.04	0.14	0.64	0.18
**G1**	Control	recovery	1/01/2011	0.71	0.04	0.07	0.61	0.28
**G1**	High	recovery	1/01/2011	0.71	0.44	0.09	0.25	0.22
**G1**	Medium	recovery	1/01/2011	0.71	0.11	0.15	0.53	0.21
**S2**	Medium	no shade	1/04/2012	0.33	0.15	0.10	0.50	0.25
**S2**	High	no shade	1/04/2012	0.30	0.07	0.08	0.73	0.12
**S2**	Low	no shade	1/04/2012	0.33	0.15	0.18	0.52	0.15
**S2**	Control	no shade	1/04/2012	0.81	0.03	0.04	0.79	0.14
**S2**	Medium	shading	1/04/2012	0.33	0.07	0.16	0.63	0.14
**S2**	High	shading	1/04/2012	0.30	0.09	0.18	0.49	0.24
**S2**	Low	shading	1/04/2012	0.33	0.07	0.15	0.63	0.14
**S2**	Control	shading	1/04/2012	0.81	0.03	0.05	0.54	0.37
**S2**	Medium	shading	1/05/2012	0.16	0.11	0.29	0.47	0.14
**S2**	High	shading	1/05/2012	0.00	0.12	0.44	0.31	0.12
**S2**	Low	shading	1/05/2012	0.45	0.14	0.28	0.41	0.17
**S2**	Control	shading	1/05/2012	0.87	0.02	0.04	0.81	0.14
**S2**	Medium	shading	1/06/2012	0.07	0.30	0.22	0.35	0.14
**S2**	High	shading	1/06/2012	0.07	0.20	0.44	0.22	0.14
**S2**	Low	shading	1/06/2012	0.63	0.11	0.24	0.44	0.20

**S2**	Control	shading	1/06/2012	0.80	0.03	0.11	0.70	0.16
**S2**	Medium	shading	1/07/2012	0.84	0.16	0.14	0.48	0.22
**S2**	High	shading	1/07/2012	0.74	0.17	0.25	0.36	0.22
**S2**	Low	shading	1/07/2012	0.84	0.16	0.13	0.51	0.21
**S2**	Control	shading	1/07/2012	0.87	0.02	0.04	0.80	0.14
**G2**	Control	no shade	1/09/2013	1.00	0.02	0.04	0.22	0.73
**G2**	High	no shade	1/09/2013	0.36	0.07	0.09	0.15	0.69
**G2**	Medium	no shade	1/09/2013	0.84	0.03	0.05	0.19	0.73
**G2**	Low	no shade	1/09/2013	0.88	0.02	0.05	0.20	0.72
**G2**	Control	shading	1/09/2013	1.00	0.02	0.04	0.35	0.59
**G2**	High	shading	1/09/2013	0.36	0.07	0.08	0.16	0.69
**G2**	Medium	shading	1/09/2013	0.84	0.03	0.05	0.33	0.58
**G2**	Low	shading	1/09/2013	0.88	0.02	0.05	0.20	0.73
**G2**	Control	shading	1/10/2013	1.00	0.02	0.03	0.22	0.73
**G2**	High	shading	1/10/2013	0.23	0.08	0.08	0.34	0.50
**G2**	Medium	shading	1/10/2013	0.81	0.02	0.04	0.13	0.82
**G2**	Low	shading	1/10/2013	0.84	0.02	0.04	0.13	0.82
**G2**	Control	shading	1/11/2013	1.00	0.02	0.03	0.22	0.73
**G2**	High	shading	1/11/2013	0.57	0.04	0.05	0.75	0.17
**G2**	Medium	shading	1/11/2013	0.97	0.02	0.03	0.22	0.73
**G2**	Low	shading	1/11/2013	0.97	0.02	0.03	0.21	0.74
**G2**	Control	recovery	1/12/2013	0.94	0.02	0.13	0.71	0.14

**G2**	High	recovery	1/12/2013	0.94	0.06	0.40	0.36	0.17
**G2**	Medium	recovery	1/12/2013	0.94	0.03	0.20	0.62	0.15
**G2**	Low	recovery	1/12/2013	0.94	0.02	0.07	0.78	0.13

Supplementary Table 13. *Zostera* Gladstone MSE

Mean Squared Error (MSE) in predicted state probabilities for biomass and for just the zero state, corresponding to the experimental designs and light scenarios (Supplementary Table 12) used for validation of *Zostera* at Gladstone, Australia[14].

	**MSE**	**MSE for Zero State**
**S1-Medium**	0.0117	0.0023
**S1-High**	0.0207	0.0019
**S1-Low**	0.0222	0.0025
**S1-Control**	0.0254	0.0027
**G1-Low**	0.0491	0.0010
**G1-Control**	0.0170	0.0005
**G1-High**	0.0163	0.0158
**G1-Medium**	0.0376	0.0019
**S2-Medium**	0.0128	0.0063
**S2-High**	0.0220	0.0180
**S2-Low**	0.0101	0.0104

**S2-Control**	0.0287	0.0047
**G2-Control**	0.0487	0.0001
**G2-High**	0.0486	0.0026
**G2-Medium**	0.0516	0.0002
**G2-Low**	0.0592	0.0001

Supplementary Table 14. *Zostera* Validation Aininkap

Data derived state probabilities for shoot density corresponding to the observational study for validation of *Zostera* at Aininkap, Japan[16]. Data was simulated from the mean, variance and number of replicates reported in the paper assuming a normal distribution.

		Zero	Low	Moderate	High
1	1/7/2000	0.0865087	0.086693	0.2610789	0.56572
2	1/8/2000	0.08155527	0.083164	0.180678	0.654603
3	1/9/2000	0.07909298	0.07803	0.1212695	0.721608
4	1/10/2000	0.08793983	0.08744	0.3326852	0.491935
5	1/11/2000	0.08722625	0.091361	0.416211	0.405202
6	1/12/2000	0.07734724	0.077265	0.1226	0.722788
7	1/05/2001	0.08346493	0.085182	0.260048	0.571305
8	1/06/2001	0.07878238	0.077406	0.1248939	0.718918
9	1/07/2001	0.09208165	0.088029	0.2532114	0.566678

Supplementary Table 15. *Zostera* Validation Aininkap Growth

Data derived state probabilities for lateral growth corresponding to the observational study of *Zostera* at Aininkap, Japan. As light and growth data do not overlap at all, these results are only indicative. Also, data was simulated from the mean, variance and number of replicates reported in the paper assuming a normal distribution.

		Zero	Low	Moderate	Fast
1	1/07/1999	0.07939551	0.07613869	0.1221899	0.72227586
2	1/08/1999	0.10734448	0.10582378	0.332772	0.45405972
3	1/09/1999	0.08662036	0.08817895	0.3388183	0.48638238
4	1/10/1999	0.09101656	0.20078176	0.5724647	0.13573697
5	1/11/1999	0.08394542	0.56386524	0.264604	0.08758531
6	1/12/1999	0.08209656	0.6551247	0.1822244	0.08055436
7	1/05/2000	0.0793447	0.07908027	0.1232366	0.71833847
8	1/06/2000	0.08056796	0.08503744	0.1868714	0.64752321

Supplementary Table 16. *Zostera* Validation Puget Sound Light

Data derived state probabilities for light saturation corresponding to the observational study for validation of *Zostera* at Puget Sound, USA.

	Month											
**Year**	Jan	Feb	Mar	Apr	May	Jun	Jul	Aug	Sep	Oct	Nov	Dec
**2008**	0.316	0.831	0.999	1	1	1	0.998	0.822	0.242	0.072	0.05	0.095
**2009**	0	0	0.614	1	1	1	1	1	0.845	0.048	0	0
**2010**	0.121	0.385	0.746	0.913	0.958	0.967	0.954	0.831	0.499	0.225	0.123	0.091
**2011**	0.1	0.98	1	1	1	1	1	0.558	0.004	0	0	0
**2012**	0.006	0.102	0.79	0.999	1	1	1	0.999	0.858	0.283	0.029	0.002
**2013**	0.001	0.015	0.472	0.997	1	1	1	1	0.908	0.247	0.017	0.001
**2014**	0.128	0.427	0.756	0.915	0.959	0.971	0.954	0.829	0.456	0.208	0.113	0.093
**2015**	0.126	0.383	0.75	0.903	0.958	0.969	0.953	0.824	0.499	0.222	0.122	0.078

**2016**	0.005	0.045	0.908	1	1	1	1	1	0.764	0.071	0.011	0.004

Supplementary Table 17. *Zostera* Validation Puget Sound Shoot Density

Data derived state probabilities for shoot density, approximated by percentage cover, corresponding to the observational study for validation of *Zostera* at Puget Sound, USA.

Date	Zero	Low	Moderate	High
**6/05/2008**	0.072438	0.149677	0.357775	0.42011
**4/07/2008**	0.055903	0.129944	0.070851	0.743302
**1/11/2008**	0.093157	0.092634	0.640781	0.173429
**10/01/2009**	0.092582	0.591963	0.268956	0.046498
**27/04/2009**	0.065966	0.261519	0.623617	0.048897
**21/07/2009**	0.058949	0.055144	0.276159	0.609748
**28/10/2009**	0.072986	0.070763	0.764368	0.091884
**31/01/2010**	0.079271	0.13023	0.749443	0.041057
**29/04/2010**	0.061094	0.104771	0.757217	0.076918
**22/07/2010**	0.060406	0.054234	0.62027	0.265091
**19/10/2010**	0.080566	0.044485	0.620756	0.254193
**20/04/2011**	0.068027	0.274965	0.60703	0.049977
**13/07/2011**	0.053903	0.051175	0.686423	0.208499
**21/10/2011**	0.064604	0.037539	0.815933	0.081923
**6/01/2012**	0.139002	0.25561	0.557357	0.048031

**9/04/2012**	0.333932	0.546304	0.069764	0.050001
**3/07/2012**	0.222524	0.341417	0.306605	0.129454
**17/10/2012**	0.068516	0.039852	0.762374	0.129258
**10/01/2013**	0.093036	0.317079	0.54558	0.044306
**29/04/2013**	0.111549	0.410294	0.422659	0.055498
**23/07/2013**	0.059924	0.056286	0.485566	0.398224
**29/04/2014**	0.065647	0.400817	0.486421	0.047115
**20/04/2015**	0.069252	0.223068	0.623322	0.084358
**2/07/2015**	0.058902	0.09022	0.171759	0.679118
**1/04/2016**	0.054641	0.810411	0.093984	0.040965
**1/07/2016**	0.062872	0.250947	0.574732	0.11145

Supplementary Table 18 Sensitivity Results

Goodness of fit R^2, mean squared error, and relative influence of node states on high, moderate, low and zero shoot density using boosted tree regression analysis of all 3024 modelled scenarios. Both the node state at the same time slice and that lagged by one time slice were used for regression. Identical results were obtained for biomass as the conditional probabilities were the same since the difference was in calibration (Methods).

```
, , t-1, R^2
Realised_Shoot_Density.High          0.9968679
Realised_Shoot_Density.Moderate      0.9971536
Realised_Shoot_Density.Low           0.9851599
Realised_Shoot_Density.Zero          0.9991339

, , t-1, MSE
Realised_Shoot_Density.High          0.021463409
Realised_Shoot_Density.Moderate      0.017410106
Realised_Shoot_Density.Low           0.048260662
Realised_Shoot_Density.Zero          0.007191633

-------High
```

Net_Change_Shoot_Density.High	*5.335928e+01*
Realised_Shoot_Density.High01	*4.089718e+01*
Baseline_Shoot_Density.Zero	*2.062456e+00*
Baseline_Shoot_Density.High	*1.033913e+00*
**Ability_to_Resist_Hazard.Strong**	**5.603795e-01**
Net_Change_Shoot_Density.Moderate	4.326686e-01
**Loss_in_Shoot_Density.Low01**	**3.595036e-01**

Baseline_Shoot_Density.Low01	1.628562e-01
Baseline_Shoot_Density.Zero01	1.401026e-01
Realised_Shoot_Density.Low01	1.342261e-01
Lateral_Growth_from_Existing_Individuals.Zero01	8.653222e-02
Baseline_Shoot_Density.High01	7.849872e-02
Loss_in_Shoot_Density.High	7.087884e-02
Net_Change_Shoot_Density.Low	6.672226e-02
Overall_Lateral_Growth.Moderate	5.691794e-02
Baseline_Shoot_Density.Moderate	5.551381e-02
Ability_to_Resist_Hazard.Strong01	5.349492e-02
Ability_to_Resist_Hazard.Weak	4.368429e-02
Overall_Lateral_Growth.Zero01	4.237797e-02
Realised_Shoot_Density.Moderate01	4.204521e-02
Net_Change_Shoot_Density.High01	3.347988e-02
Lateral_Growth_from_Existing_Individuals.Moderate	3.164295e-02
Lateral_Growth_from_Existing_Individuals.Fast01	2.973660e-02
Loss_in_Shoot_Density.Moderate	1.861663e-02
Rate_of_Recovery_in_Shoot_Density.Zero	1.636245e-02
Net_Change_Shoot_Density.Moderate01	1.431099e-02
Ability_to_Resist_Hazard.Weak01	1.176730e-02
Lateral_Growth_from_Existing_Individuals.Zero	1.174344e-02
Loss_in_Shoot_Density.Zero01	7.405370e-03
Overall_Lateral_Growth.Zero	7.297430e-03
Rate_of_Recovery_in_Shoot_Density.Low	6.697626e-03
Recruitment_Rate_from_Seeds.Absent	6.268049e-03
Ability_to_Recover.High	6.142934e-03
Genera_Presence.Zostera	5.820007e-03
Net_Change_Shoot_Density.Low01	5.539045e-03

Realised_Shoot_Density.Zero01	4.902473e-03
Genera_Presence.Halophila	4.822939e-03
Rate_of_Recovery_in_Shoot_Density.High	4.079906e-03
Location_Type.TransitoryTropicalDeepSubTidal	3.725340e-03
Overall_Lateral_Growth.Slow	3.709149e-03
Time_of_Year.Apr	3.422741e-03
Overall_Lateral_Growth.Moderate01	2.917166e-03
Ability_to_Recover.Zero	2.591962e-03
Loss_in_Shoot_Density.Zero	2.359913e-03
Rate_of_Recovery_in_Shoot_Density.High01	2.347578e-03
Lateral_Growth_from_Existing_Individuals.Fast	2.155447e-03
Lateral_Growth_from_Existing_Individuals.Slow	1.970404e-03
Seed_Density.Low	1.661902e-03
Physiological_Status_of_Plants.Medium	1.540323e-03
Accumulated_Light.BelowSaturation01	1.177963e-03
Rate_of_Recovery_in_Shoot_Density.Zero01	7.755685e-04
Recruitment_Rate_from_Seeds.Low01	7.314848e-04
Baseline_Shoot_Density.Low	6.843749e-04
Ability_to_Recover.Low01	4.589626e-04
Overall_Lateral_Growth.Slow01	4.581411e-04
Recruitment_Rate_from_Seeds.Low	4.575129e-04
Physiological_Status_of_Plants.Good	3.411871e-04
Loss_in_Shoot_Density.Moderate01	3.250640e-04
Loss_in_Shoot_Density.High01	3.150503e-04

------ Moderate

	rel.inf
Realised_Shoot_Density.Moderate01	*7.012839e+01*

Net_Change_Shoot_Density.Moderate	*2.195382e+01*
Baseline_Shoot_Density.Zero	*3.292514e+00*
Realised_Shoot_Density.Zero01	*1.394822e+00*
Realised_Shoot_Density.High01	7.964770e-01
Net_Change_Shoot_Density.Low01	5.425305e-01
**Loss_in_Shoot_Density.Low**	**3.708496e-01**
**Lateral_Growth_from_Existing_Individuals.Moderate**	**2.292180e-01**
**Loss_in_Shoot_Density.Zero**	**2.043145e-01**
**Overall_Lateral_Growth.Slow**	**1.546288e-01**
Baseline_Shoot_Density.Moderate	1.534010e-01
Baseline_Shoot_Density.Zero01	1.103600e-01
Genera_Presence.Halophila	7.438884e-02
Baseline_Shoot_Density.High	7.209984e-02
Ability_to_Resist_Hazard.Strong	7.018651e-02
Net_Change_Shoot_Density.Zero	6.783916e-02
Overall_Lateral_Growth.Moderate	5.091905e-02
Lateral_Growth_from_Existing_Individuals.Zero01	4.474377e-02
Rate_of_Recovery_in_Shoot_Density.Moderate	3.967780e-02
Realised_Shoot_Density.Low01	3.120176e-02
Ability_to_Resist_Hazard.Weak01	2.817139e-02
Ability_to_Resist_Hazard.Weak	2.742077e-02
Rate_of_Recovery_in_Shoot_Density.High01	2.572099e-02
Baseline_Shoot_Density.Low	2.099970e-02
Ability_to_Resist_Hazard.Strong01	1.442481e-02
Seed_Density.Absent01	1.266542e-02
Ability_to_Recover.Low	9.413151e-03
Accumulated_Light.BelowSaturation	8.761908e-03
Baseline_Shoot_Density.Low01	7.826547e-03

Net_Change_Shoot_Density.Moderate01	7.316115e-03
Recruitment_Rate_from_Seeds.Absent	6.178684e-03
Physiological_Status_of_Plants.Medium	6.114491e-03
Overall_Lateral_Growth.Moderate01	5.400607e-03
Lateral_Growth_from_Existing_Individuals.Slow	4.792267e-03
Net_Change_Shoot_Density.High01	4.185449e-03
Ability_to_Recover.Zero	4.018252e-03
Rate_of_Recovery_in_Shoot_Density.Low	3.527839e-03
Net_Change_Shoot_Density.High	3.037344e-03
Loss_in_Shoot_Density.Low01	1.950853e-03
Accumulated_Light.AboveSaturation	1.883805e-03
Ability_to_Recover.Zero01	1.775450e-03
Overall_Lateral_Growth.Zero01	1.729535e-03
Net_Change_Shoot_Density.Low	1.430491e-03
Overall_Lateral_Growth.Slow01	1.304096e-03
Rate_of_Recovery_in_Shoot_Density.Zero01	1.255784e-03
Ability_to_Recover.High01	9.985211e-04
Genera_Presence.Zostera	9.498531e-04
Rate_of_Recovery_in_Shoot_Density.Low01	9.113864e-04
Baseline_Shoot_Density.High01	8.289067e-04
Baseline_Shoot_Density.Moderate01	6.511267e-04
Loss_in_Shoot_Density.Moderate	5.767892e-04
Physiological_Status_of_Plants.Good	5.700763e-04
Physiological_Status_of_Plants.Poor	4.421936e-04
Recruitment_Rate_from_Seeds.Low01	3.832716e-04

-------- Low

	rel.inf
Overall_Lateral_Growth.Fast	***28.822707463***
Net_Change_Shoot_Density.Low	*18.441314301*
Realised_Shoot_Density.Low01	*13.384150440*
Lateral_Growth_from_Existing_Individuals.Fast	***12.942418334***
Recruitment_Rate_from_Seeds.High01	***4.652577810***
Baseline_Shoot_Density.Zero	*3.952328684*
Rate_of_Recovery_in_Shoot_Density.High01	***3.724907657***
Net_Change_Shoot_Density.Moderate	*3.656523849*
Net_Change_Shoot_Density.High	*2.578259923*
Ability_to_Recover.High01	***1.165383831***
Baseline_Shoot_Density.Low	*1.085167875*
Baseline_Shoot_Density.Moderate01	*1.028357996*
Time_of_Year.Jan	0.647138747
Baseline_Shoot_Density.Moderate	0.511802846
Location_Type.PersistentTemperateShallowSubTidal	0.448676570
Seed_Density.High	0.416367547
Baseline_Shoot_Density.High	0.391051706
Seed_Density.High01	0.316603106
Rate_of_Recovery_in_Shoot_Density.Low01	0.271707366
Ability_to_Recover.Zero01	0.174815446
Baseline_Shoot_Density.High01	0.140078695
Overall_Lateral_Growth.Fast01	0.126430570
Overall_Lateral_Growth.Slow	0.103507005
Overall_Lateral_Growth.Moderate	0.095025311
Ability_to_Recover.Zero	0.094816358
Recruitment_Rate_from_Seeds.Absent01	0.092088587

Recruitment_Rate_from_Seeds.High	0.088631842
Net_Change_Shoot_Density.Zero	0.082453761
Physiological_Status_of_Plants.Poor	0.072875291
Seed_Density.Low01	0.067333287
Recruitment_Rate_from_Seeds.Absent	0.057352476
Seed_Density.Absent01	0.056539077
Seed_Density.Low	0.042280166
Rate_of_Recovery_in_Shoot_Density.Zero	0.037961760
Ability_to_Resist_Hazard.Strong01	0.036094848
Overall_Lateral_Growth.Zero	0.033085947
Ability_to_Resist_Hazard.Weak01	0.025210777
Recruitment_Rate_from_Seeds.Low01	0.019575671
Rate_of_Recovery_in_Shoot_Density.Moderate	0.016517231
Ability_to_Recover.High	0.016249132
Realised_Shoot_Density.Moderate01	0.012897891
Ability_to_Resist_Hazard.Weak	0.011708688
Loss_in_Shoot_Density.High01	0.009520122
Loss_in_Shoot_Density.Low	0.007920196
Net_Change_Shoot_Density.High01	0.007864919
Baseline_Shoot_Density.Low01	0.007270568
Ability_to_Recover.Low	0.006393861
Loss_in_Shoot_Density.Moderate01	0.004542889
Location_Type.PersistentTropicalInterTidal	0.002790186
Net_Change_Shoot_Density.Moderate01	0.002568238
Seed_Density.Absent	0.001995264
Rate_of_Recovery_in_Shoot_Density.High	0.001787158
Overall_Lateral_Growth.Moderate01	0.001675679
Physiological_Status_of_Plants.Medium	0.001663777

Net_Change_Shoot_Density.Low01 0.001636241

Realised_Shoot_Density.Zero01 0.001395034

-------- Zero

	rel.inf
Net_Change_Shoot_Density.Zero	*4.099055e+01*
Ability_to_Resist_Hazard.Strong	***1.956807e+01***
Ability_to_Resist_Hazard.Weak	***1.932620e+01***
Baseline_Shoot_Density.Zero	*5.302873e+00*
Lateral_Growth_from_Existing_Individuals.Zero	***5.065761e+00***
Realised_Shoot_Density.Zero01	*4.686381e+00*
Overall_Lateral_Growth.Zero	***1.984153e+00***
Baseline_Shoot_Density.High	*1.329372e+00*
Baseline_Shoot_Density.Low	5.050709e-01
Realised_Shoot_Density.Low01	2.173845e-01
Net_Change_Shoot_Density.Low	1.910452e-01
**Ability_to_Recover.Low**	**1.775381e-01**
Baseline_Shoot_Density.Moderate	1.586104e-01
**Overall_Lateral_Growth.Moderate**	**1.367580e-01**
**Ability_to_Recover.Zero**	**1.240032e-01**
Time_of_Year.Jan	8.904678e-02
Time_of_Year.Apr	5.761536e-02
Lateral_Growth_from_Existing_Individuals.Moderate	2.106293e-02
Loss_in_Shoot_Density.Zero	7.188485e-03
Rate_of_Recovery_in_Shoot_Density.Low	6.677425e-03
Lateral_Growth_from_Existing_Individuals.Slow	6.634215e-03

Recruitment_Rate_from_Seeds.Absent	6.246182e-03
Net_Change_Shoot_Density.Zero01	5.029287e-03
Rate_of_Recovery_in_Shoot_Density.High	4.432931e-03
Recruitment_Rate_from_Seeds.Low	4.022922e-03
Baseline_Shoot_Density.High01	3.192927e-03
Baseline_Shoot_Density.Zero01	2.580845e-03
Recruitment_Rate_from_Seeds.High	2.495939e-03
Loss_in_Shoot_Density.Moderate	2.410083e-03
Loss_in_Shoot_Density.Low01	2.257989e-03
Rate_of_Recovery_in_Shoot_Density.Zero	2.072948e-03
Recruitment_Rate_from_Seeds.Absent01	1.983675e-03
Baseline_Shoot_Density.Moderate01	1.681979e-03
Lateral_Growth_from_Existing_Individuals.Zero01	1.234198e-03
Seed_Density.High01	9.626691e-04
Accumulated_Light.BelowSaturation	9.190081e-04
Net_Change_Shoot_Density.Low01	7.728338e-04
Net_Change_Shoot_Density.High	7.146104e-04
Ability_to_Recover.High	6.917191e-04
Overall_Lateral_Growth.Zero01	6.199214e-04
Ability_to_Recover.Zero01	4.503371e-04
Physiological_Status_of_Plants.Poor	4.063006e-04
Overall_Lateral_Growth.Moderate01	4.039523e-04
Overall_Lateral_Growth.Fast	3.982606e-04
Accumulated_Light.AboveSaturation	3.507864e-04
Accumulated_Light.AboveSaturation01	2.846104e-04
Physiological_Status_of_Plants.Medium	2.409123e-04
Net_Change_Shoot_Density.Moderate01	2.199682e-04
Accumulated_Light.BelowSaturation01	1.946507e-04

Loss_in_Shoot_Density.High	1.810838e-04
Net_Change_Shoot_Density.Moderate	1.586981e-04
Loss_in_Shoot_Density.Moderate01	1.556213e-04
Rate_of_Recovery_in_Shoot_Density.Zero01	1.225547e-04
Net_Change_Shoot_Density.High01	1.171515e-04

Supplementary Table 19 Baseline Check

Expert and literature based check of predicted baseline patterns of seagrass population, growth and phenology for each of the 25 sites in the global map. The main expert consulted was Dr Kathryn McMahon (for credentials, refer to Supplementary Table 5). NP = Data not yet published.

Port	Genera	Meadow	Growing Season	Flowering and/or Seed Season	Notes	Check	Reference
**James Price Point, Australia**	*Halophila*	Annual	Dry season, reflected by model	Seed always present and highest Oct-Jan; model predicts Oct-Mar. Seed lowest in growing season from Jun-Aug, captured in model.	Highest cover in Oct-Nov, reflected in model	Correct	[2]
**Jurien Bay, Australia**	*Amphibolis*	Enduring	Growth validated against data	Viviparous seedlings present March to June[13]	Shoot density, biomass, growth rate and physiological status validated against data	Correct	[4]
**Singapore**	*Halophila*	Enduring	Jun-Sep	May-Nov	Seed is all year round at many tropical locations.	Correct	[5]
**Mombasa, Kenya**	*Halophila*	Enduring	NA	NA	Similar to Singapore as are results except it is in Southern hemisphere	Correct	-
**Hay Point, Australia**	*Halophila*	Transitory	NA	NA	Biomass was validated against	Correct	[8]

					data		
**Hay Point Inshore, Australia**	*Halophila*	Annual	NA	NA	See Hay Point	Correct	[8]
**Adelaide Waters, Australia**	*Amphibolis*	Enduring	Peak growth in spring	Viviparous seedling present Sept-Feb. Model predicts peak seed formation Oct-Mar.	Declined to zero following dredging.	Correct	[10,30,31]
**Adelaide Waters site 2, Australia**	*Amphibolis*	Enduring	Peak growth in spring	Viviparous seedling present Sept-Feb. Model predicts peak seed formation Oct-Mar.	-	Correct	[10,30,31]
**Cockburn Sound, Australia**	*Amphibolis*	Enduring	Spring-summer	Viviparous seedling present	-	Correct	[12]
**Geraldton, Australia**	*Amphibolis*	Enduring	See Jurien Bay	See Jurien Bay	Similar dynamics to Jurien Bay	Correct	[4]
**Gladstone, Australia**	*Zostera*	Enduring	Growth was validated against data	NA	Biomass was validated against data	Correct	[14]
**Aininkap, Japan**	*Zostera*	Enduring	Growth was validated against data	Nov-Feb; model predicts Oct-Dec - sufficiently close for available data	Shoot density was validated against data	Correct	[16]
**Moreton Bay (western side**	*Zostera*	Enduring	NA	NA	Similar dynamics as Gladstone in	Correct	[14]

**Waterloo Bay) , Australia**					model and in real life		
**Red Sea Phosphate Terminal, Israel**	*Halophila*	Enduring	Meadow extends to up to 45m depth in Summer and only 30m depth in Winter	Expect similar seed pattern to James Price Point	Looks reasonable, like Singapore or India (Feb-Apr, Med Jul-Aug)	Correct	[32]
**Red Sea Open Sea, Israel**	*Halophila*	Enduring	Same as above	Same as above	Same as above	Correct	[32]
**Red Sea Navy, Israel**	*Halophila*	Enduring	Same as above	Same as above	Same as above;	Correct	[32]
**Point Franklin, Port of Melbourne, Australia**	*Amphibolis*	Enduring	NA	NA	Similar dynamics and environments as Cockburn sound	Correct	[12]
**Mud Island, Port of Melbourne, Australia**	*Zostera*	Enduring	NA	flowering mid spring to early summer coincides with seed density increase in model	-	Correct	[19]
**Cameron's Bight, Port of Melbourne, Australia**	*Zostera*	Enduring	NA	Same as above	-	Correct	[19]
**Kobbefjord, Greenland**	*Zostera*	Implied Enduring	Growth highest in	seeds observed Aug; 1-2mo to produce;	-	Correct	[20]

		Olesen	Summer	coincides with peak seed period in model			
**Ameralik, Greenland**	*Zostera*	Same as above	Growth highest in Summer	Same as above	-	Correct	[20]
**Kapisillit, Greenland**	*Zostera*	Same as above	Growth highest in Summer	Same as above	-	Correct	[20]
**Roskilde, Denmark**	*Zostera*	Enduring	-	-	More similar to Aininkap than Greenland	Correct	[16,22]
**Waquoit Bay, USA**	*Zostera*	Enduring	Highest growth May-July, model is Mar-July	Late summer seed, corresponds to peak in model	Lowest Nov, highest May-Jun; close enough fit, ~1-2months off	Correct	[23]
**Puget Sound Site A, USA**	*Zostera*	Enduring	Spring-Summer	NA	Shoot density seasonal pattern validated against data.	Correct	NP
**Salt River Canyon, St Croix**	*Halophila*	Transitory	Seasonal declines Oct-Feb	NA	Biomass seasonal pattern validated against data.	Correct	[25]
**Puget Sound Site C, USA**	*Zostera*	Enduring	Spring-Summer	NA	Shoot density seasonal pattern validated against data.	Correct	NP

Supplementary Text: Modelling Assumptions and Supplementary References

Assumes constant connectivity effects

Assumes only stresses are light related (zero sediment quality stress, burial stress, grazing or other stresses)

Assumes probability of above saturation light during dredging period to be the minimum of baseline probability for that month and the light stress scenario of 0%, 25%, 50% or 75%

Assumes negligible effects of competition e.g. form macroalgae

Assumes negligible effects of epiphytes

Assumes adequate nutrition

References Used In Supplementary Materials

1 Pearl, J. *Probabilistic Reasoning in Intelligent Systems.* (Morgan Kaufmann, 1988).

2 Hovey, R. K. *et al.* Strategy for assessing impacts in ephemeral tropical seagrasses. *Marine pollution bulletin* **101**, 594-599 (2015).

3 Dennison, W. C. *et al.* Assessing water quality with submersed aquatic vegetation. *BioScience* **43**, 86-94 (1993).

4 McMahon, K., Lavery, P. S. & Mulligan, M. Recovery from the impact of light reduction on the seagrass \emph{Amphibolis griffithii}, insights for dredging management. *Marine Pollution Bulletin* **62**, 270-283 (2011).

5 Yaakub, S. M., Chen, E., Bouma, T. J., Erftemeijer, P. L. & Todd, P. A. Chronic light reduction reduces overall resilience to additional shading stress in the seagrass Halophila ovalis. *Marine*

pollution bulletin **83**, 467-474 (2014).

6 McClanahan, T. R. Seasonality in East Africa's coastal waters. *Marine ecology progress series. Oldendorf* **44**, 191-199 (1988).

7 Lee, K.-S., Park, S. R. & Kim, Y. K. Effects of irradiance, temperature, and nutrients on growth dynamics of seagrasses: a review. *Journal of Experimental Marine Biology and Ecology* **350**, 144-175 (2007).

8 York, P. H. *et al.* Dynamics of a deep-water seagrass population on the {Great Barrier Reef}: annual occurrence and response to a major dredging program. *Scientific Reports* **5:13167** (2015).

9 McCormack, C. *et al.* Interim Report: Deepwater Seagrass Dynamics - Light requirements, seasonal change and mechanisms of recruitment for deepwater seagrasses. 26pp (Centre for Tropical Water & Aquatic Ecosystem Research (TropWATER), James Cook University, Cairns, 2013).

10 Collings, G., Miller, D., O'Loughlin, E., Cheshire, A. & Bryars, S. Turbidity and reduced light responses of the meadow forming seagrasses Amphibolis and Posidonia, from the Adelaide metropolitan coastline, Publication No. RD01/0208-17. (Adelaide Coastal Waters Study Steering Committee, Adelaide, 2006).

11 Lavery, P., McMahon, K., Mulligan, M. & Tennyson, A. Interactive effects of timing, intensity and duration of experimental shading on Amphibolis griffithii. *Marine Ecology* **394**, 21-33 (2009).

12 De Roach, R. Fremantle Port Inner Harbour and Channel Deepening - Reclamation at Rous Head and Offshore Placement of Dredged Material. Compliance Assessment Report: Phase 1 Works. Report No. 816_003/2, (Fremantle Ports 2010).

13 McMahon, K. & Lavery, P. The responses of Amphibolis griffithii to reduced light availability. *Final report on the Strategic Research Fund for the Marine Environment (SRFME) Collaborative Research Project: ecophysiology of benthic primary producers. Centre for Marine Ecosystems Research, Edith Cowan University, Joondalup, Western Australia* (2008).

14 Chartrand, K. M., Bryant, C. V., Carter, A. B., Ralph, P. J. & Rasheed, M. A. Light thresholds to prevent dredging impacts on the Great Barrier Reef seagrass, Zostera muelleri ssp. capricorni.

Frontiers in Marine Science **3**, 106 (2016).

15 Collier, C. J. *et al.* Thresholds for morphological response to light reduction for four tropical seagrass species. *Ecological Indicators* **67**, 358-366, doi:http://dx.doi.org/10.1016/j.ecolind.2016.02.050 (2016).

16 Watanabe, M., Nakaoka, M. & Mukai, H. Seasonal variation in vegetative growth and production of the endemic Japanese seagrass Zostera asiatica: a comparison with sympatric Zostera marina. *Botanica Marina* **48**, 266-273 (2005).

17 Longstaff, B. J. *Investigations into the light requirements of seagrasses in northeast Australia* Ph.D. thesis, University of Queensland, (2003).

18 Schwarz, A.-M. & Hellblom, F. The photosynthetic light response of Halophila stipulacea growing along a depth gradient in the Gulf of Aqaba, the Red Sea. *Aquatic Botany* **74**, 263-272 (2002).

19 Edmunds, M., Pickett, P. & Stewart, K. Port Phillip Bay Channel Deepening Project Supplementary Environmental Effects Statement **-** Marine Ecology Specialist Studies. (Australian Marine Ecology Pty Ltd, 2006).

20 Olesen, B., Krause-Jensen, D., Marbà, N. & Christensen, P. B. Eelgrass Zostera marina in subarctic Greenland: dense meadows with slow biomass turnover in cold waters. *Marine Ecology Progress Series* **518**, 107-121 (2015).

21 Marsh, J. A., Dennison, W. C. & Alberte, R. S. Effects of temperature on photosynthesis and respiration in eelgrass (Zostera marina L.). *Journal of Experimental Marine Biology and Ecology* **101**, 257-267 (1986).

22 Staehr, P. A. & Borum, J. Seasonal acclimation in metabolism reduces light requirements of eelgrass (Zostera marina). *Journal of Experimental Marine Biology and Ecology* **407**, 139-146 (2011).

23 Hauxwell, J., Cebrian, J. & Valiela, I. Light dependence of Zostera marina annual growth dynamics in estuaries subject to different degrees of eutrophication. *Aquatic botany* **84**, 17-25 (2006).

24 Thom, R. M., Southard, S. L., Borde, A. B. & Stoltz, P. Light requirements for growth and survival of eelgrass (Zostera marina L.) in Pacific Northwest (USA) estuaries. *Estuaries and Coasts* **31**, 969-980 (2008).

25 Williams, S. L. Disturbance and recovery of a deep-water Caribbean seagrass bed. *Marine ecology progress series. Oldendorf* **42**, 63-71 (1988).

26 McMahon, K., Collier, C. & Lavery, P. S. Identifying robust bioindicators of light stress in seagrasses: A meta-analysis. *Ecological Indicators* **30**, 7-15 (2013).

27 Kilminster, K. *et al.* Unravelling complexity in seagrass systems for management: Australia as a microcosm. *Science of the Total Environment* **534**, 97-109 (2015).

28 Kendrick, G. A. *et al.* The central role of dispersal in the maintenance and persistence of seagrass populations. *BioScience* **62**, 56-65 (2012).

29 Wu, P. P.-Y., Mengersen, K., McMahon, K., Kendrick, G. A. & Caley, M. J. in *MODSIM2015 21st Int. Congress on Modelling and Simulation.* 1282-1288 (MSSANZ, 2015).

30 Nayar, S., Collings, G., Miller, D. & Bryars, S. Nutrient fluxes in the meadow forming seagrasses Posidonia and Amphibolis from the Adelaide metropolitan coast. Report No. Publication No. RD01/0208-18, (Adelaide, 2006).

31 Westphalen, G. *et al.* A review of seagrass loss on the Adelaide metropolitan coastline. Report No. Publication No. RD04/0073, (Adelaide Coastal Waters Study Steering Committee, Adelaide, 2004).

32 Sharon, Y. *et al.* Photosynthetic responses of Halophila stipulacea to a light gradient. II. Acclimations following transplantation. *Aquatic Biology* **7**, 153-157 (2009).
